# Perylenequione Derivatives with Anticancer Activities Isolated from the Marine Sponge-Derived Fungus, *Alternaria* sp. SCSIO41014

**DOI:** 10.3390/md16080280

**Published:** 2018-08-14

**Authors:** Xiaoyan Pang, Xiuping Lin, Pei Wang, Xuefeng Zhou, Bin Yang, Junfeng Wang, Yonghong Liu

**Affiliations:** 1 CAS Key Laboratory of Tropical Marine Bio-resources and Ecology, Guangdong Key Laboratory of Marine Materia Medica, RNAM Center for Marine Microbiology, South China Sea Institute of Oceanology, Chinese Academy of Sciences, Guangzhou 510301, China; luckygirlpxy@163.com (X.P.); xiupinglin@hotmail.com (X.L.); xfzhou@scsio.ac.cn (X.Z.); yangbin@scsio.ac.cn (B.Y.); 2 University of Chinese Academy of Sciences, Beijing 100049, China; 3 Key Laboratory of Biology and Genetic Resources of Tropical Crops, Ministry of Agriculture, Institute of Tropical Bioscience and Biotechnology, Chinese Academy of Tropical Agricultural Sciences, Haikou 571101, China; wangpei@itbb.org.cn

**Keywords:** sponge-derived fungus, *Alternaria* sp., perylenequinone derivatives, X-ray single crystal diffraction, cytotoxic activity, antibacterial

## Abstract

Seven new secondary metabolites classified as two perylenequinone derivatives (**1** and **2**), an altenusin derivative (**3**), two phthalide racemates (**4** and **5**), and two phenol derivatives (**6** and **7**), along with twenty-one known compounds (**8**–**28**) were isolated from cultures of the sponge-derived fungus, *Alternaria* sp. SCSIO41014. The structures and absolute configurations of these new compounds (**1**–**7**) were determined by spectroscopic analysis, X-ray single crystal diffraction, chiral-phase HPLC separation, and comparison of ECD spectra to calculations. Altertoxin VII (**1**) is the first example possessing a novel 4,8-dihydroxy-substituted perylenequinone derivative, while the phenolic hydroxy groups have commonly always substituted at C-4 and C-9. Compound **1** exhibited cytotoxic activities against human erythroleukemia (K562), human gastric carcinoma cells (SGC-7901), and hepatocellular carcinoma cells (BEL-7402) with IC_50_ values of 26.58 ± 0.80, 8.75 ± 0.13, and 13.11 ± 0.95 μg/mL, respectively. Compound **11** showed selectively cytotoxic activity against K562, with an IC_50_ value of 19.67 ± 0.19 μg/mL. Compound **25** displayed moderate inhibitory activity against *Staphylococcus aureus* with an MIC value of 31.25 μg/mL.

## 1. Introduction

**Figure 1:**
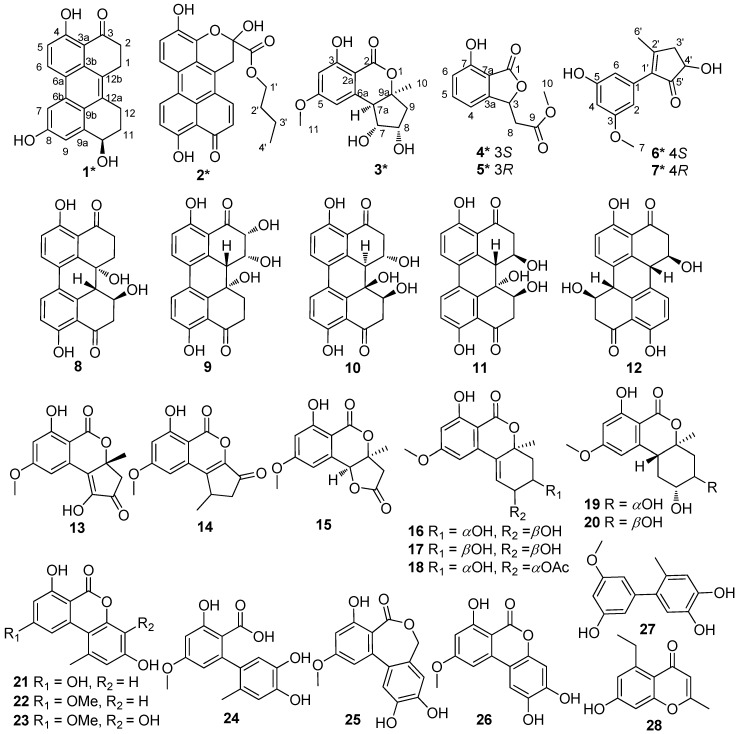
Chemical structures of compounds **1**–**28**.

Perylenequinone derivatives are secondary metabolites characterized by a conjugated aromatic pentacyclic dione, which are mainly derived from fungi [1,2]. Hypocrellins, which are the typical perylenequinone derivatives isolated from fungi *Hypocrella bambusae* and *Shiraia bambusicola*, have been studied for their light-induced antitumor and antiviral activities [3]. Furthermore, many perylenequinone derivatives isolated from *Alternaria* sp. showed phytotoxicity, as well as antimicrobial and anticancer activities [4,5,6,7]. Sponge-derived fungi are one of the richest sources of many structurally unique and biologically active secondary metabolites among marine sources [8]. As part of our ongoing research for bioactive natural products from sponge-derived fungi [9,10,11,12,13], the fungus *Alternaria* sp. SCSIO41014 was studied. Seven new (**1**–**7**) and twenty-one known (**8**–**28**) compounds (Figure 1) were isolated from the culture extract of the fungus *Alternaria* sp. SCSIO41014. Altertoxin VII (**1**) is the first example possessing a novel 4,8-dihydroxy-substituted perylenequinone derivative, while the phenolic hydroxy groups have always commonly substituted at C-4 and C-9. Herein, we describe the structure elucidation and bioactivity evaluation of new compounds **1**–**7**.

## 2. Results and Discussion

### 2.1. Structural Elucidation

**Figure 2:**
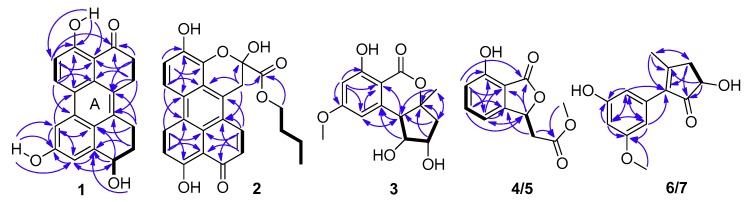
COSY “H—H” and key HMBC “H→C” correlations of compounds **1**–**7**.

**Figure 3:**
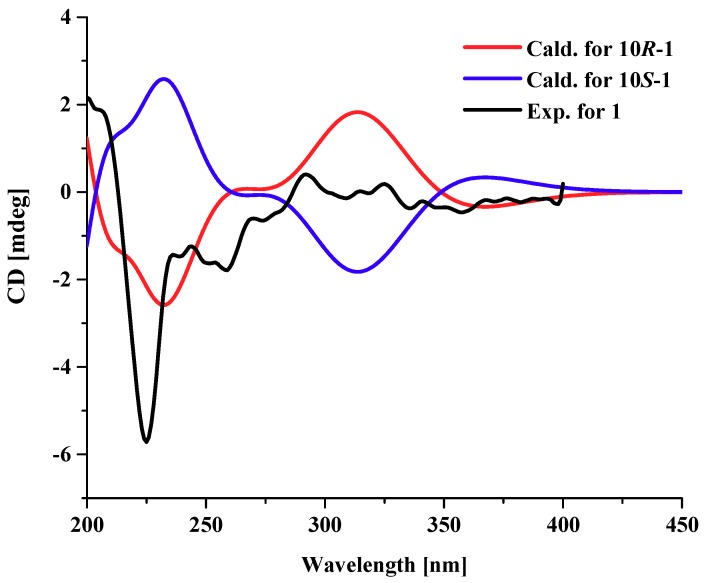
Comparison between calculated and experimental electronic circular dichroism (ECD) spectra of compound **1**.

Compound **1** was obtained as a dark red powder. Its molecular formula was established as C_20_H_16_O_4_ by ^13^C NMR data and the high resolution electrospray ionization mass spectroscopy (HRESIMS) [M + Na]^+^ peak at *m*/*z* 343.0939 (calculated for C_20_H_15_O_4_Na, 343.0941), indicating thirteen degrees of unsaturation. Its UV spectrum showed maxima at 323 and 368 nm, which suggested that **1** featured a polycyclic aromatic hydrocarbons system. Its ^1^H NMR data (Table 1) showed two *ortho* aromatic protons (*δ*_H_ 8.79, d, *J* = 9.5 Hz, H-6 and 7.16, d, *J* = 9.0 Hz, H-5), two *meta* aromatic protons (*δ*_H_ 7.82, d, *J* = 2.0 Hz, H-7 and 7.30, d, *J* = 1.5 Hz, H-9), an oxygenated methine (*δ*_H_ 4.82, dd, *J* = 9.0, 3.0 Hz, H-10), and three hydroxy protons (*δ*_H_ 13.27, brs, OH-4; 9.87, brs, OH-8; 5.48, brs, OH-10). The striking downfield shift of OH-4 is a typical feature of a strong hydrogen bond, which is in accordance with the downfield shift of C-3 (*δ*_C_ 204.0) observed in the ^13^C NMR spectrum. The ^13^C NMR (DEPT) (Table 1) data also exhibited twenty carbon signals, including four sp^3^ methylenes, one oxygenated sp^3^ methine (*δ*_C_ 67.4, C-10), four sp^2^ methines (*δ*_C_ 133.1, C-6; 116.2, C-5; 114.4, C-9; 104.4, C-7), ten sp^2^ non-protonated carbons, and one carbonyl. Besides the presence of two benzene rings, a double bond, and a carbonyl, three degrees of unsaturation were left, which indicated that there were another three rings in the structure of **1**. Half of its NMR data were identical to those of 4,9-dihydroxy-1,2,11,12-tetrahydroperylene-3,10-quinone [14], while compound **1** was not a symmetric structure, and the other half of the data had two obvious differences. One carbonyl and two *ortho* aromatic protons in 4,9-dihydroxy-1,2,11,12-tetrahydroperylene-3,10-quinone were replaced by an oxygenated methine and two *meta* aromatic protons, respectively. The differences were testified by the cross-peaks of H_2_-1 (*δ*_H_ 3.25)/H_2_-2 (*δ*_H_ 2.92), of H-5/H-6, and of OH-10/H-10/H_2_-11 (*δ*_H_ 2.18 and 1.88)/H_2_-12 (*δ*_H_ 3.20 and 3.00) in the ^1^H-^1^H correlation spectroscopy (COSY) spectrum (Figure 2), and the correlations from: H-7 and H-9 to C-8; from H-10 and H_2_-11 to C-9a; from H-7, H-9, H-10 and H_2_-12 to C-9b; from H-9 to C-7 and C-10; and from H_2_-11 and H_2_-12 to C-12a in the heteronuclear multiple bond correlation (HMBC) spectrum (Figure 2). Furthermore, it is allowed for the linkage of ring A (Figure 2) by the correlations from H_2_-1 to C-12a; from H-6 to C-6b; from H-7 to C-6a; and from H_2_-12 to C-12b in the HMBC spectrum. The attachment of the three hydroxy groups was further confirmed by the correlations from: OH-4 to C-3a, C-4 and C-5; from OH-8 to C-7, C-8 and C-9; and from OH-10 to C-9a (Figure 2). Therefore, the planar structure of **1** was established as 4,8,10-trihydroxy-1,2,11,12-tetrahydroperylene-3-quinone. Its nuclear Overhauser effect spectroscopy (NOESY) spectrum displayed cross-peaks of H_2_-1/H_2_-12 and of H-6/H-7, which also support the conjectural structure above. The absolute configuration of **1** was established based on comparison of its experimental electronic circular dichroism (ECD) curve with the calculated ECD curve of the 10*R* and the 10*S* model at the B3LYP/6-31G(d,p) level in Gaussian 03, and the former was in accordance with the experimental one (Figure 3). Thus, the absolute structure of **1** was defined as (10*R*)-4,8,10-trihydroxy-1,2,11,12-tetrahydroperylene-3-quinone, and named altertoxin VII.

Compound **2** was obtained as a dark red powder. Its molecular formula was assigned as C_24_H_20_O_7_ based on its [M + Na]^+^ ion at *m*/*z* 443.1110 and [M + H]^+^ ion at *m*/*z* 421.1284 in the HRESIMS spectrum, indicating fifteen degrees of unsaturation. Analysis of its ^1^H NMR and ^13^C NMR data revealed that the structural features of **2** were similar to those of xanalteric acid II [5], except for the presence of an oxygenated *n*-butyl group (*δ*_C/H_ 65.1/4.09, CH_2_-1′; 30.0/1.46, CH_2_-2′; 18.3/1.14, CH_2_-3′; 13.4/0.71, CH_3_-4′), which was confirmed by its COSY cross-peaks of H_2_-1′/H_2_-2′/H_2_-3′/H_3_-4′. The location of the oxygenated *n*-butyl group was ascertained by the HMBC correlation from H_2_-1′ to C-13. Based on the HMBC correlations (Figure 2), the planar structure of **2** was determined as butyl 2,4,9-trihydroxy-10-oxo-2,10-dihydro-1*H*-phenaleno[1,2,3-*de*] chromene-2-carboxylate and named butyl xanalterate. Due to the existence of the cyclic hemi-ketal, compound **2** was not stable under the protic solvent condition, meaning that the absolute configuration was uncertain.

**Figure 4:**
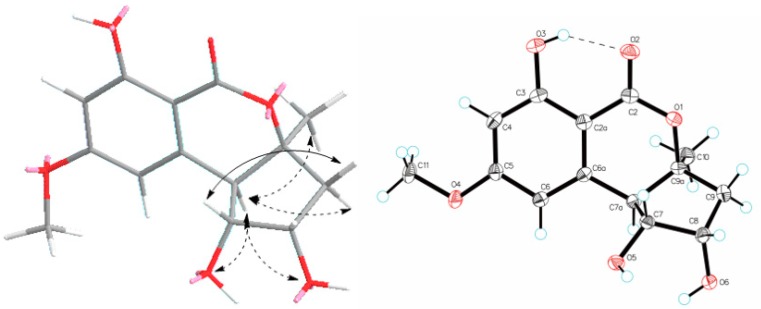
Key NOESY correlations and ORTEP drawing of compound **3**.

Compound **3** possessed a molecular formula of C_14_H_16_O_6_ on the basis of its NMR data and the HRESIMS ion peak at *m*/*z* 303.0844 [M + Na]^+^, indicating seven indices of hydrogen deficiency. The ^1^H NMR spectrum (Table 2) exhibited two aromatic protons (*δ*_H_ 6.52, brs, H-6; 6.43, brs, H-4); three methines (*δ*_H_ 4.20, ddd, *J* = 7.0, 5.5, 2.5 Hz, H-8; 3.90, dd, *J* = 10.5, 5.0 Hz, H-7; 3.07, d, *J* = 10.0 Hz, H-7a); one methoxy group (*δ*_H_ 3.88, s, H_3_-11); and a methyl singlet (*δ*_H_ 1.47, s, H_3_-10). The ^13^C NMR and HSQC spectra indicated the presence of 14 carbons, including two methyls (*δ*_C_ 56.2, C-11; 25.7, C-10), one sp^3^ methylene, three sp^3^ methines (two oxygenated at *δ*_C_ 80.1, C-7; 71.2, C-8), two sp^2^ methines (*δ*_C_ 101.0, C-4; 108.9, C-6), four sp^2^ non-protonated carbon, and an ester carbonyl (*δ*_C_ 170.0, C-2). Because one benzene ring and one carbonyl accounted for five degrees of unsaturation, double ring fragment was required for the structure of **3**. Its NMR data were similar to those of dihydroaltenuenes A (**19**) [15], the obvious differences being the disappearance of a methylene signal (*δ*_C_ 28.1) and the downfield shift of the oxygenated methine in **3**, which indicated that a fragment of the six-membered ring in **19** was replaced by a five-membered ring in **3**. The deduction was confirmed by following COSY correlations of H-7a/H-7/H-8/H_2_-9, as well as HMBC correlations, from H-7 and H-7a to C-6a; from H-6, H_2_-9 and H_3_-10 to C-7a; and from H-8, H_2_-9 and H_3_-10 to C-9a (Figure 2). The NOESY spectrum in DMSO-*d*_6_ (Figure 4) exhibited the cross-peaks of H-7a to H-9*α*, H_3_-10, OH-7, and OH-8, and also of H-9*β* to H-7, which suggested that CH_3_-10, H-7a, OH-7, and OH-8 were on the same side. The Cu Kα radiation for the X-ray diffraction experiment with the refined Flack parameter of −0.02(7) allowed the assignment of the absolute configuration of all the stereogenic centers in **3** as 7*R*, 7a*R*, 8*S*, and 9a*S*. Thus, the absolute structure of **3** was unambiguously elucidated and named nordihydroaltenuenes A.

**Figure 5:**
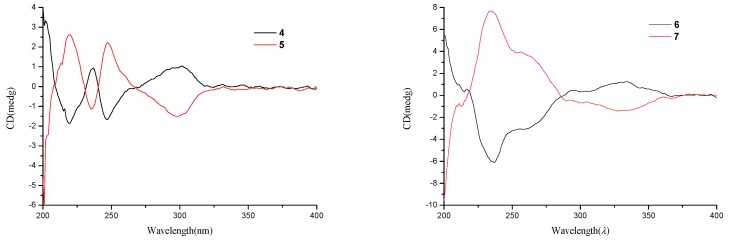
Experimental ECD spectra of compounds **4**–**7**.

Compounds **4** and **5** were initially believed to be a single compound, which was obtained as a yellow oil. The molecular formula was established as C_11_H_15_O_5_ based on the HRESIMS and ^13^C NMR data. A literature survey suggested that the ^1^H NMR and ^13^C NMR data (Table 2) closely resembled those of isoochracinic acid [16], except for the presence of a methoxy group (*δ*_C/H_ 52.5/3.69, C-10) and an upfield shift of 1.3 ppm of the carbonyl (*δ*_C_ 171.6, C-9), which indicated the carboxyl in the isoochracinic acid was methylated. The deduction was confirmed by the HMBC correlation of H_3_-10 to C-9. The planar structure of **4**/**5** was further established by the COSY and HMBC correlations (Figure 2). The optical rotation was small ([*α*${]}_{D}^{25}$ +1.6) and the value of the ECD spectrum close to zero, suggesting that compounds **4** and **5** belonged to a racemate. Using a chiral-phase column (Daicel Chiraloak IC-3, 250 × 4.6 mm, 5 μm), the racemate was resolved to two enantiomers, **4** and **5**, whose scale was nearly 1:1 (Figure S30). The optical rotation of **4** ([*α*${]}_{D}^{25}$ −21, *c* 0.1, MeOH) and **5** ([*α*${]}_{D}^{25}$ +26, *c* 0.1, MeOH), as well as the ECD spectrum (Figure 5), were opposite to each other. In general, the 3*R*-phthalides displayed a positive optical rotation, while 3*S*-phthalides had a negative one [17,18,19,20]. Therefore, the absolute configuration of compound **4** was proposed as 3*S* and named (*S*)-isoochracinate A1, while compound **5** was proposed as 3*R*, and named (*R*)-isoochracinate A2.

Compounds **6** and **7,** obtained as a yellow powder, were also believed to be a single compound at first. The HRESIMS spectrum supported a molecular formula of C_13_H_14_O_4_, requiring 7 degrees of unsaturation. The ^1^H NMR and ^13^C NMR data (Table 2) of **6/7** were almost identical to those of 4′-(*S*)-(3,5-dihydroxyphenyl)-4′-hydroxy-6′-methylcyclopent-1′-en-5′-one isolated from *Penicillium* sp. HN29-3B1 [21], indicating that **6/7** were a structural analog, except for the presence of a methoxy group (*δ*_C/H_ 55.7, C-7). The deduction was confirmed by the HMBC correlation (Table 2) from H_3_-7 to C-3. Analyzed by a chiral-phase column (Phenomenex Lux Cellulose-2, 250 × 4.6 mm, 5 μm), compounds **6/7** were found to be stereoisomeric mixture, whose mass ratio was about 1:3.5 (Figure S39). The single compound **6** ([*α*${]}_{D}^{25}$ +3.1, *c* 0.1, MeOH) and **7** ([*α*${]}_{D}^{25}$ −7.2, *c* 0.1, MeOH) were isolated, and they had opposite ECD spectra (Figure 5). The optical rotation of **6** was consistent with that of 4′-(*S*)-(3,5-dihydroxyphenyl)-4′-hydroxy-6′-methylcyclopent-1′-en-5′-one [21], indicating that they had the same *S* configuration at C-4′, opposite to that of **7**. Thus, the absolute structure of **6** was determined as 4′-(*S*)-(3-Methoxy-5-hydroxyphenyl)-4′-hydroxy-6′-methylcyclopent-1′-en-5′-one and named (*S*)-alternariphent A1, while the absolute structure of **7** was determined as 4′-(*R*)-(3-Methoxy-5-hydroxyphenyl)-4′-hydroxy-6′-methylcyclopent-1′-en-5′-one and named (*R*)-alternariphent A2.

By comparing NMR data with the values in literature, the structures of the twenty-one known compounds were identified as altertoxin I (**8**) [22], 7-epi-8-hydroxyaltertoxin (**9**) [6], stemphytriol (**10**) [23], 6-epi-stemphytriol (**11**) [6], stemphyperylenol (**12**) [23,24], (*R*)-1,6-dihydroxy-8-methoxy-3a-methyl-3,3a-dihydrocyclopenta[*c*]iso- chromene-2,5-dione (**13**) [25], 1-deoxyrubralactone (**14**) [25], 6-hydroxy-8-methoxy-3a-methyl-3a,9b-dihydro-3*H*-furo[3,2-c]isochromene-2,5-dione (**15**) [26], altenuene (**16**) [27], 4′-epialtenuene (**17**) [28], (−)-(2*R*,3*R*,4a*R*)-altenuene-3-acetoxy ester (**18**) [29], dihydroaltenuenes A (**19**) [15], 3-epi-dihydroaltenuene A (**20**) [30], alternariol (**21**) [31], alternariol monomethyl ether (**22**) [32], 3′-hydroxyalternariol-5-*O*-methyl ether (**23**) [28], altenusin (**24**) [33], alterlactone (**25**) [28], altenuisol (**26**) [34], 5′-methoxy-6-methyl-biphenyl-3,4,3′-triol (**27**) [26], and 2,5-dimethyl-7-hydroxychromone (**28**) [35], respectively.

### 2.2. Biological Activity

The cytotoxic activities of compounds **1**, **2**, and **8**–**12** against human erythroleukemia (K562), human gastric carcinoma cells (SGC-7901), and hepatocellular carcinoma cells (BEL-7402) were evaluated using the CCK-8 method, and paclitaxel was used as a positive control with IC_50_ values of 0.18 ± 0.20, 0.89 ± 0.15, and 0.54 ± 0.20 μg/mL, respectively. Among the tested compounds, compound **1** exhibited cytotoxic activity against the K562, SGC-7901, and BEL-7402 cell lines with IC_50_ values of 26.58 ± 0.80, 8.75 ± 0.13, and 13.11±0.95 μg/mL, respectively. Compound **11** showed selectively cytotoxic activity against K562 with an IC_50_ value of 19.67 ± 0.19 μg/mL. All compounds were tested for their antibacterial activities against *Staphylococcus aureus.* Compounds **10** and **25** with 50 μg/disc displayed an inhibition zone with a diameter of about 21 and 15 mm, respectively (Figure S1). Furthermore, their minimum inhibitory concentrations (MIC) were tested, and the MIC value of compound **25** was 31.25 μg/mL, while compound **10** showed more than 500 μg/mL—perhaps due to its poor solubility. Ampicillin was used as a positive control with an MIC value of 6.25 μg/mL.

## 3. Materials and Methods

### 3.1. General Experimental Procedures

HRESIMS data were recorded on a maXis Q-TOF mass spectrometer in a positive ion mode (Bruker, Fällanden, Switzerland). 1D and 2D NMR spectra were measured on an AV 500 MHz NMR spectrometer (Bruker, Fällanden, Switzerland) with TMS as an internal standard. Chemical shifts were given as *δ* values, with *J* values reported in Hz. Optical rotations were measured using a MCP-500 polarimeter (Anton, Austria). UV spectra were recorded on a UV-2600 UV-Vis spectrophotometer (Shimadzu, Japan). X-ray diffraction intensity data were collected on an XtalLAB PRO single-crystal diffractometer using Cu Kα radiation (Rigaku, Japan). ECD spectrum was measured with a Chirascan circular dichroism spectrometer (Applied Photophysics, Surrey, UK). HPLC was performed on a Hitachi Primaide with the YMC ODS SERIES column (YMC-Pack ODS-A, YMC Co. Ltd., Kyoto, Japan, 250 × 10 mm I.D., S-5 μm, 12 nm) and chiral-phase column (Phenomenex Lux Cellulose-2 column, 4.6 mm × 25 mm and Daicel Chiraloak IC-3 column, 4.6 mm × 25 mm). Column chromatography (CC) was carried out on silica gel (200–300 mesh, Jiangyou Silica Gel Development Co., Yantai, China), Sephadex LH-20 (40–70 μm, Amersham Pharmacia Biotech AB, Uppsala, Sweden), and YMC Gel ODS-A (12 nm, S-50 μm YMC Co. Ltd., Kyoto, Japan). Spots were detected on TLC under UV light or by heating after spraying with the mixed solvent of saturated vanillin and 5% H_2_SO_4_ in EtOH. The TLC plates with silica gel GF254 (0.4–0.5 mm, Qingdao Marine Chemical Factory, Qingdao, China) were used for analysis and for the preparatives.

### 3.2. Fungal Material

The fungal strain SCSIO41014 was obtained from a *Callyspongia* sp. sponge, which was collected from the sea area near Xuwen County, Guangdong Province, China. The producing strain was stored on MB agar (malt extract 15 g, agar 16 g, sea salt 10 g, water 1 L, pH 7.4–7.8) slants at 4 °C and deposited at the Key Laboratory of Tropical Marine Bio-resources and Ecology, Chinese Academy of Science. The ITS1-5.8S-ITS2 sequence region (508 base pairs, GenBank accession No. MH444654) of strain SCSIO41014 was amplified by PCR, and DNA sequencing showed it shared a significant homology to several species of *Setosphaeria*. The 508 base pairs of the ITS sequence had a 99% sequence identity to that of the *Alternaria alternata* strain SCAU091 (GenBank accession No. MF061753.1). It was thus designated as a member of *Alternaria* sp. and named *Alternaria* sp. SCSIO41014.

### 3.3. Fermentation and Extraction

The strain SCSIO41014 was cultured in 100 mL flasks (×59) each containing 10 mL seed medium (malt extract: 15 g, sea salt: 2.5 g, distilled water: 1 L, pH 7.4–7.8) at 27 °C on a rotary shaker (172 rpm) for 48 h. The mass fermentation of this fungus was carried out at 25 °C for 32 days using a rice medium (rice: 200 g/flask, sea salt: 2.5 g/flask, tap water: 200 mL/flask) in the 1 L flasks (×59). The flasks were incubated statically at 25 °C under the normal day and night cycle. After 32 days, cultures were soaked in acetone (400 mL/flask), mashed into small pieces, and vibrated with ultrasound for 20 min. Then the acetone solution was evaporated under reduced pressure to afford an aqueous solution, which was extracted with ethyl acetate (EtOAc) three times. Concurrently, the rice residue was extracted with EtOAc in order to make another EtOAc solution. Both of the EtOAc solutions were combined and concentrated under reduced pressure to produce a crude extract. The extract was then suspended in MeOH and partitioned with equal volumes of petroleum ether (PE). Finally, the MeOH solution was concentrated under reduced pressure to obtain a reddish-brown extract (78.0 g).

### 3.4. Isolation and Purification

The reddish-brown extract was subjected to silica gel CC, which was eluted with a CH_2_Cl_2_ and MeOH mixed solvent in a step gradient (100:0–3:1, *v*/*v*) and separated into seven fractions (Fr-1–Fr-7). Fr-1 (3.4 g) was subjected to reversed-phase C-18 MPLC with MeOH/H_2_O (35:65–100:0, *v*/*v*) to get four fractions (Fr-1-1–Fr-1-4). Fr-1-1 was directly separated by semi-preparative HPLC (75% MeOH/H_2_O, 1.6 mL/min) to produce **22** (5.4 mg, t_R_ = 25 min). Fr-1-2 was purified by semi-preparative HPLC (42% CH_3_CN/H_2_O, 2 mL/min) to yield **13** (26.0 mg, t_R_ = 22.0 min) and **14** (5.6 mg, t_R_ = 34.0 min). Fr-1-3 was separated by semi-preparative HPLC (30% CH_3_CN/H_2_O, 2 mL/min) to yield **15** (17.8 mg, t_R_ = 43.0 min). Fr-1-4 was purified by semi-preparative HPLC (28% CH_3_CN/H_2_O, 2 mL/min) to afford raceme **4/5** (97.3 mg, t_R_ = 18.0 min), part of which was further separated by HPLC with a chiral-phase column (Daicel Chiraloak IC-3 column, 4.6 mm × 25 mm, eluent *n*-hexane/*iso*-propanol, 65:35 *v*/*v*, 1 mL/min) to obtain **4** (3.3 mg, t_R_ = 15.6 min) and **5** (2.8 mg, t_R_ = 17.0 min). Fr-3 (15.8 g) was subjected to silica gel CC eluted with a PE and acetone mixed solvent in a step gradient (10:1–0:1, *v*/*v*) to gain five fractions (Fr-3-1–Fr-3-5). Fr-3-3 (0.94 g) was subjected to a Sephadex LH-20 column, eluted with MeOH and further purified by semi-preparative HPLC, to produce **18** (7.6 mg, 40% CH_3_CN/H_2_O, 2 mL/min, t_R_ = 30.0 min), **23** (8.0 mg, 45% CH_3_CN/H_2_O, 2 mL/min, t_R_ = 22.0 min), and **28** (22.5 mg, 45% CH_3_CN/H_2_O, 2 mL/min, t_R_ = 24.0 min). Fr-3-4 (7.3 g) was subjected to reversed-phase C-18 MPLC with MeOH/H_2_O (10:90–100:0, *v*/*v*) to get three fractions (Fr-3-4-1–Fr-3-4-3). Fr-3-4-1 was subjected to a Sephadex LH-20 column eluted with MeOH to gain four parts. One part was purified by a preparative thin-layer chromatography, using CH_2_Cl_2_/MeOH (10:1) as a developing solvent to afford **16** (42.6 mg, Rf = 0.6) and **19** (11.3 mg, Rf = 0.7). Other parts were separated by semi-preparative HPLC to yield **3** (3.5 mg, 60% MeOH/H_2_O, 2 mL/min, t_R_ = 13.5 min), **9** (4.8 mg, 60% MeOH/H_2_O, 2 mL/min, t_R_ = 15.8 min), **10** (10.3 mg, 60% MeOH/H_2_O, 2 mL/min, t_R_ = 19.0 min), and **25** (104.0 mg, 34% CH_3_CN/H_2_O, 2 mL/min, t_R_ = 20.0 min). Fr-3-4-2 was subjected to a Sephadex LH-20 column, eluted with MeOH and purified by semi-preparative HPLC, to yield **11** (5.2 mg, 56% MeOH/H_2_O, 2 mL/min, t_R_ = 26.0 min), **12** (13.1 mg, 35% CH_3_CN/H_2_O, 2 mL/min, t_R_ = 21.4 min), and **21** (20.6 mg, 40% CH_3_CN/H_2_O, 2 mL/min, t_R_ = 21.0 min). Fr-3-4-3 was subjected to silica gel CC with a PE and EtOAc mixed solvent in a step gradient (5:1–1:1, *v*/*v*), and then purified by semi-preparative HPLC to yield **26** (7.0 mg, 30% CH_3_CN/H_2_O, 2 mL/min, t_R_ = 26.4 min). Fr-3-5 (1.1 g) was subjected to a Sephadex LH-20 column eluted with MeOH, and then purified by semi-preparative HPLC to yield **2** (6.7 mg, 55% CH_3_CN/H_2_O, 2 mL/min, t_R_ = 32.0 min) and **8** (3.9 mg, 48% CH_3_CN/H_2_O, 2 mL/min, t_R_ = 18.0 min). Fr-4 (8.4 g) was subjected to silica gel CC eluted with a PE and acetone mixed solvent in a step gradient (10:1–0:1, *v*/*v*) to gain three fractions (Fr-4-1–Fr-4-3). Fr-4-2 (5.1 g) was subjected to a Sephadex LH-20 column eluted with MeOH, then subjected to reversed-phase C-18 MPLC with MeOH/H_2_O (10:90–100:0, *v*/*v*), and further purified by semi-preparative HPLC, to obtain **1** (17.4 mg, 38% CH_3_CN/H_2_O, 2 mL/min, t_R_ = 28.2 min), **17** (34.7 mg, 56% MeOH/H_2_O, 2 mL/min, t_R_ = 21.5 min), **20** (10.6 mg, 28% CH_3_CN/H_2_O, 2 mL/min, t_R_ = 13.0 min), and the stereoisomeric mixture **6/7** (13.2 mg, 15% CH_3_CN/H_2_O, 2 mL/min, t_R_ = 23.6 min). Part of the mixture **6/7** was separated by HPLC with a chiral-phase column (Phenomenex Lux Cellulose-2, 4.6 mm × 25 mm, eluent *n*-hexane/*iso*-propanol, 40:60 *v*/*v*, 1 mL/min) to afford **6** (1.5 mg, t_R_ = 7.4 min) and **7** (5.2 mg, t_R_ = 8.4 min). Fr-5 (13.1 g) was subjected to silica gel CC eluted with a PE and acetone mixed solvent in a step gradient (10:1–0:1, *v*/*v*) to gain three fractions (Fr-5-1–Fr-5-3). Fr-5-2 (3.7 g) was subjected to a Sephadex LH-20 column eluted with MeOH, which was then subjected to reversed-phase C-18 MPLC with MeOH/H_2_O (10:90–100:0, *v*/*v*), and further purified by semi-preparative HPLC, to yield **27** (8.2 mg, 52% MeOH/H_2_O, 2 mL/min, t_R_ = 17.6 min) and **24** (21.9 mg, 52% MeOH/H_2_O, 2 mL/min, t_R_ = 28.0 min).

### 3.5. Spectral Data

*Altertoxin VII* (**1**): dark red powder; [*α*${]}_{D}^{25}$ −9.0 (*c* 0.02, MeOH); UV(MeOH) *λ*_max_(log *ε*) 226 (3.06), 253 (3.36), 323 (2.68), 368 (2.45) nm; ECD (0.38 mM, MeOH) *λ*_max_ (Δ*ε*) 204 (+0.92), 224 (–2.77) and 253 (−0.87) nm; ^1^H NMR (DMSO-*d*_6_, 500 MHz) and ^13^C NMR (DMSO-*d*_6_, 125 MHz), Table 1; HRESIMS *m*/*z* 343.0939 [M + Na]^+^ (calcd for C_20_H_16_NaO_4_, 343.0941).

*Butyl xanalterate* (**2**): dark red powder; [*α*${]}_{D}^{25}$ −42 (*c* 0.02, MeOH); ^1^H NMR (DMSO-*d*_6_, 500 MHz) and ^13^C NMR (DMSO-*d*_6_, 125 MHz), Table 1; HRESIMS *m*/*z* 443.1110 [M + Na]^+^ (calcd for C_24_H_20_NaO_7_, 443.1101) and 421.1284 [M + H]^+^ (calcd for C_24_H_21_O_7_, 421.1282).

*Nordihydroaltenuenes A* (**3**), colourless oil, [*α*${]}_{D}^{25}$ +113 (*c* 0.07, MeOH); UV(MeOH) *λ*_max_(log *ε*) 211 (3.26), 269 (2.97), 307 (2.64) nm; ECD (0.24 mM, MeOH) *λ*_max_ (Δ*ε*) 207 (–15.34), 232 (+7.75), 246 (+0.43) and 271 (+6.89) nm; ^1^H NMR (CD_3_OD, 500 MHz) and ^13^C NMR (CD_3_OD, 125 MHz), Table 2; HRESIMS *m*/*z* 303.0844 [M + Na]^+^ (calcd for C_14_H_16_NaO_6_, 303.0839).

*Isoochracinate A* (**4** and **5**): yellow oil; [*α*${]}_{D}^{25}$ +1.6 (*c* 0.1, MeOH); UV (MeOH) *λ*_max_ (log *ε*) 207 (3.34), 233 (2.59), 300 (2.38) nm; ^1^H NMR (CD_3_OD, 500 MHz) and ^13^C NMR (CD_3_OD, 125 MHz), Table 2; HRESIMS *m*/*z* 223.0606 [M + H]^+^ (calcd for C_11_H_11_O_5_, 223.0607); (*S*)-isoochracinate A1 (**4**), [*α*${]}_{D}^{25}$ −21 (*c* 0.1, MeOH), ECD (0.45 mM, MeOH) *λ*_max_ (Δ*ε*) 219 (–1.25), 237 (+0.64), 247 (–1.12) and 301 (+0.70) nm; (*R*)-isoochracinate A2 (**5**), [*α*${]}_{D}^{25}$ +26 (*c* 0.1, MeOH), ECD (0.32 mM, MeOH) λ_max_ (Δ*ε*) 220 (+1.77), 235 (–0.76), 247 (+1.50) and 300 (–0.99) nm.

*Alternariphent A* (**6** and **7**): yellow powder; [*α*${]}_{D}^{25}$ −4.6 (*c* 0.1, MeOH); UV(MeOH) *λ*_max_(log *ε*) 206 (2.37) nm; ^1^H NMR (CD_3_OD, 500 MHz) and ^13^C NMR (CD_3_OD, 125 MHz), Table 2; HRESIMS *m*/*z* 257.0787 [M + Na]^+^ (calcd for C_13_H_14_NaO_4_, 257.0784). (*S*)-Alternariphent A1 (**6**), [*α*${]}_{D}^{25}$ +3.1 (*c* 0.1, MeOH), ECD (0.64 mM, MeOH) *λ*_max_ (Δ*ε*) 211 (+0.47), 237 (–2.90), 259 (–1.46) and 334 (+0.59) nm; (*R*)-Alternariphent A2 (**7**), [*α*${]}_{D}^{25}$ −7.2 (*c* 0.1, MeOH), ECD (0.51 mM, MeOH) λ_max_ (Δ*ε*) 213 (–0.59), 233 (+4.53), 256 (+2.34) and 328 (–0.84) nm.

### 3.6. X-ray Crystal Structure Analysis

Crystallographic data for compound *nordihydroaltenuenes A* (**3**) were collected on a Rigaku XtaLAB PRO single-crystal diffractometer using Cu K*α* radiation. The structure of **3** was solved by direct methods (SHELXS 97), expanded using difference Fourier techniques, and refined by using the full-matrix least-squares calculation. The non-hydrogen atoms were refined anisotropically, and hydrogen atoms were fixed at calculated positions. Crystallographic data for the structure **3** has been deposited with the Cambridge Crystallographic Data Centre with the supplementary publication number CCDC-1847869. Copies of the data can be obtained free of charge from the CCDC at www.ccdc.cam.ac.uk.

*Crystal data for***3**: Moiety formula: C_14_H_16_O_6_ (*M*_W_ = 280.27), colourless block, crystal size = 0.2 × 0.1 × 0.1 mm^3^, orthorhombic, space group P2_1_2_1_2_1_; unit cell dimensions: *a* = 6.75000(10) Å, *b* = 8.05910(10) Å, *c* = 22.8555(3) Å, *V* = 1243.31(3) Å^3^, *Z* = 4, *ρ*_calcd_ = 1.497 g cm^−3^, *T* = 100.00(10) K, *μ*(Cu Kα) = 0.995 mm^−1^. A total of 5819 reflections were measured with 2434 independent reflections (*R*_int_ = 0.0209, *R*_sigma_ = 0.0222). Final *R* indices (I > 2*σ* (*I*)): *R*_1_ = 0.0277, w*R*_2_ = 0.0730. Final R indexes (all date): *R*_1_ = 0.0281, w*R*_2_ = 0.0734, Flack parameter = −0.02(7). Largest diff. peak and hole = 0.15 and −0.19 eÅ^−3^.

### 3.7. ECD Calculations

The theoretical calculations of new compound **1** was performed by using the density functional theory (DFT) as carried out in the Gaussian 03 [36]. Conformational analysis was initially conducted by using SYBYL-X 2.0 software. All ground-state geometries were optimized at the B3LYP/6-31G(d) level. TDDFT at B3LYP/6-31G(d) was employed to calculate the electronic excitation energies and rotational strengths in methanol [37,38]. Solvent effects of methanol solution were evaluated at the same DFT level by using the SCRF/PCM method [39].

### 3.8. Biological Assays

#### 3.8.1. Antibacterial Activity Assay

Compounds **1**–**28** were tested for antibacterial activities against *Staphylococcus aureus* using the agar filter-paper diffusion method. Then, compounds which had an inhibition zone were evaluated in 96-well plates using a modified version of the broth microdilution method, and ampicillin was used as a positive control [40].

#### 3.8.2. Antitumor Activity Assay

The in vitro cytotoxic activities against the three tumor cell lines (K562, SGC-7901 and BEL-7402) were assessed by the CCK-8 method, and the positive control was taxol [41].

## 4. Conclusions

Seven new structurally diverse polyketide derivatives (**1**–**7**), along with 21 known compounds (**8**–**28**), were isolated from cultures of the sponge-derived fungus, *Alternaria* sp. SCSIO41014. The structures and absolute configurations of these new compounds (**1**–**7**) were determined by spectroscopic analysis, X-ray single crystal diffraction, chiral separation, and comparison of ECD spectra to the calculations. Altertoxin VII (**1**) was the first example to possess a novel 4,8-dihydroxy substituted perylenequinone derivative, while the phenolic hydroxy groups always commonly substituted at C-4 and C-9. Compound **1** exhibited cytotoxic activities against the K562, SGC-7901, and BEL-7402 cell lines, with IC_50_ values of 26.58 ± 0.80, 8.75 ± 0.13, and 13.11 ± 0.95 μg/mL, respectively. Compound **11** showed selectively cytotoxic activity against K562 with an IC_50_ value of 19.67 ± 0.19 μg/mL. Compound **25** displayed moderate inhibitory activity against *Staphylococcus aureus,* with an MIC value of 31.25 μg/mL.

## Figures and Tables

**Table 1:** ^1^H NMR and ^13^C NMR data for compounds **1** and **2** in DMSO-*d*_6_ (500, 125 MHz).

No.	1		No.	2	
	*δ*_C_, type	*δ*_H_ (*J* in Hz)		*δ*_C_, type	*δ*_H_ (*J* in Hz)
1	23.2, CH_2_	3.25 td (7.0, 2.0)	1	33.9, CH_2_	4.03 d (16.5) 3.91 d (17.0)
2	36.4, CH_2_	2.92 t (7.5)	2	95.5, C	
3	204.8, C		3		
3a	110.7, C		3a	137.8, C	
3b	131.6, C		3b	118.3, C	
4	161.9, C		4	142.8, C	
5	116.2, CH	7.16 d (9.0)	5	122.2, CH	7.51 d (9.0)
6	133.1, CH	8.79 d (9.5)	6	115.6, CH	8.35 d (9.0)
6a	120.7, C		6a	124.4, C	
6b	130.8, C		6b	120.3, C	
7	104.4, CH	7.82 d (2.0),	7	133.5, CH	9.08 d (9.5)
8	156.0, C		8	118.0, CH	7.40 d 9.0
9	114.4, CH	7.30 d (1.5)	9	165.6, C	
9a	142.1, C		9a	111.3, C	
9b	120.1, C		9b	124.6, C	
10	67.4, CH	4.82 dd (9.0, 3.0)	10	188.2, C	
11	31.5, CH_2_	2.18 dq (10.0, 4.5) 1.88 dtd (12.5, 9.5, 4.5)	11	125.9, CH	6.94 d (10.0)
12	24.3, CH_2_	3.20 dt (17.0, 5.5) 3.00 ddd (16.5, 10.0, 5.0)	12	139.0, CH	8.63 d (10.0)
12a	133.4, C		12a	119.9, C	
12b	121.4, C		12b	136.5, C	
OH-4		13.27 brs	13	168.6, C	
OH-8		9.87 brs	1′	65.1, CH_2_	4.09 td (6.5, 2.0)
OH-10		5.48 brs	2′	30.0, CH_2_	1.46 qui (7.5)
			3′	18.3, CH_2_	1.14 sex (7.5)
			4′	13.4, CH_3_	0.71 t (7.5)
			OH-2		9.76 brs
			OH-4		8.14 brs
			OH-9		15.12 brs

**Table 2:** ^1^H NMR and ^13^C NMR data for compounds **3**–**7** in CD_3_OD (500, 125 MHz).

No.	3		No.	4/5		No.	6/7	
	*δ*_C_, type	*δ*_H_ (*J* in Hz)		*δ*_C_, type	*δ*_H_ (*J* in Hz)		*δ*_C_, type	*δ*_H_ (*J* in Hz)
2	170.0, C		1	171.2, C		1	134.6, C	
2a	100.1, C		3	78.3, CH	5.80 (dd, 8.0, 4.5)	2	109.8, CH	6.31 overlap
3	167.8, C		3a	152.1, C		3	162.2, C	
4	101.0, CH	6.43 brs	4	113.9, CH	6.99 (d, 7.5)	4	101.9, CH	6.35 t (2.5)
5	167.8, C		5	137.8, CH	7.52 (t, 8.0)	5	159.5, C	
6	108.9, CH	6.52 brs	6	117.0, CH	6.88 (d, 8.0)	6	107.2, CH	6.32 overlap
6a	143.1, C		7	158.2, C		7	55.7, CH_3_	3.75 s
7a	51.8, CH	3.07 d (10.0)	7a	112.4, C		1’	139.1, C	
7	80.1, CH	3.90 dd (10.5, 5.0)	8	40.0, CH_2_	2.76 dd (16.5, 8.0) 3.07 dd (17.0, 4.5)	2’	171.9, C	
8	71.2, CH	4.20 ddd (7.0, 5.5, 2.5)	9	171.6, C		3’	41.9, CH_2_	3.05 dd (18.0, 6.5) 2.50 ddd (18.0, 3.0, 1.5)
9*β* 9*α*	47.8, CH_2_	2.60 dd (15.5, 7.0) 2.04 dd (15.5, 1.5)	10	52.5, CH_3_	3.69 s	4’	72.6, CH	4.30 dd (7.0, 3.0)
9a	89.4, C					5’	208.8, C	
10	25.7, CH_3_	1.47 s				6’	18.5, CH_3_	2.18 s
11	56.2, CH_3_	3.88 s						

## Supplementary Materials

The following materials are available online at http://www.mdpi.com/1660-3397/16/8/280/s1. The 16S rRNA gene sequences data of *Alternaria* sp. SCSIO41014, the 1D and 2D NMR spectra, HRESIMS for the new compounds **1**–**7**, and X-ray crystallographic files of compound **3** (in CIF format).
