# Supplementary material for: Perylenequione Derivatives with Anticancer Activities Isolated from the Marine Sponge-Derived Fungus, Alternaria sp. SCSIO41014
Source: Mar Drugs. 2018 Aug 14;16(8):280. doi: 10.3390/md16080280 (PMC6117713; doi:10.3390/md16080280)
Supplement: Supplementary file 1 [file marinedrugs-16-00280-s001.pdf]

marine drugs

MDPI

Perylenequione Derivatives with Anticancer Activities Isolated from the Marine Sponge-Derived Fungus, *Alternaria* sp. SCSIO41014

Xiaoyan Pang [1,2], Xiuping Lin [1], Pei Wang [3], Xuefeng Zhou [1], Bin Yang [1], Junfeng Wang [1,*] and Yonghong Liu [1,2,*]

[1] CAS Key Laboratory of Tropical Marine Bio-resources and Ecology, Guangdong Key Laboratory of Marine Materia Medica, RNAM Center for Marine Microbiology, South China Sea Institute of Oceanology, Chinese Academy of Sciences, Guangzhou 510301, China; luckygirlpxy@163.com (X.P.); xiupinglin@hotmail.com (X.L.); xfzhou@scsio.ac.cn (X.Z.); yangbin@scsio.ac.cn (B.Y.)

[2] University of Chinese Academy of Sciences, Beijing 100049, China

[3] Key Laboratory of Biology and Genetic Resources of Tropical Crops, Ministry of Agriculture, Institute of Tropical Bioscience and Biotechnology, Chinese Academy of Tropical Agricultural Sciences, Haikou 571101, China; wangpei@itbb.org.cn

* Correspondence: wangjunfeng@scsio.ac.cn (J.F.W.); yonghongliu@scsio.ac.cn (Y.H.L.); Tel.: +86-020-8902-3174 (J.F.W.); +86-020-8902-3244 (Y.H.L.)

Table of Contents

Original data of antibacterial activity assay.

Figure S1 Pictures of inhibition zones in the filter paper diffusion test

Table S1 The growth conditions of the *Staphylococcus aureus* after add different dilution concentration of compounds **10**, **25** and ampicillin.

The 16S rRNA gene sequences data of *Alternaria* sp. SCSIO41014.

Theory and Calculation Details of compound 1.

Figure S2. ^{1}H NMR spectrum of **1** in DMSO-d_6.

Figure S3. ^{13}C NMR spectrum of **1** in DMSO-d_6.

Figure S4. DEPT NMR spectrum of **1** in DMSO-d_6.

Figure S5. ^{1}H-^{1}H COSY spectrum of **1** in DMSO-d_6.

Figure S6. HSQC spectrum of **1** in DMSO-d_6.

Figure S7. HMBC spectrum of **1** in DMSO-d_6.

Figure S8. NOESY spectrum of **1** in DMSO-d_6.

Figure S9. HRESIMS spectrum of **1** in CD_3OD

Figure S10. ^{1}H NMR spectrum of **2** in DMSO-d_6.

Figure S11. ^{13}C NMR spectrum of **2** in DMSO-d_6.

Figure S12. DEPT NMR spectrum of **2** in DMSO-d_6.

Figure S13. ^{1}H-^{1}H COSY spectrum of **2** in DMSO-d_6.

Figure S14. HSQC spectrum of **2** in DMSO-d_6.

Figure S15. HMBC spectrum of **2** in DMSO-d_6.

Figure S16. HRESIMS spectrum of **2** in CD_3OD.

Figure S17. ^{1}H NMR spectrum of **3** in CD_3OD.

Figure S18. ^{13}C NMR spectrum of **3** in CD_3OD.

Figure S19. ^{1}H-^{1}H COSY spectrum of **3** in CD_3OD.

Figure S20. HSQC spectrum of **3** in CD_3OD.

Figure S21. HMBC spectrum of **3** in CD_3OD.

Figure S22. NOESY spectrum of **3** in CD_3OD.

Figure S23. HRESIMS spectrum of **3** in CD_3OD.

Figure S24. ^{1}H NMR spectrum of **4/5** in CD_3OD.

Figure S25. ^{13}C NMR spectrum of **4/5** in CD_3OD.

Figure S26. ^{1}H-^{1}H COSY spectrum of **4/5** in CD_3OD.

Figure S27. HSQC spectrum of **4/5** in CD_3OD.

Figure S28. HMBC spectrum of **4/5** in CD_3OD.

Figure S29. HRESIMS spectrum of **4/5** in CD_3OD.

Figure S30. Chiral-phase HPLC analyses of compounds **4** and **5**.

Figure S31. ^{1}H NMR spectrum of **6/7** in CD_3OD.

Figure S32. ^{13}C NMR spectrum of **6/7** in CD_3OD.

Figure S33. DEPT NMR spectrum of **6/7** in CD_3OD.

Figure S34. COSY spectrum of **6/7** in CD_3OD.

Figure S35. HSQC spectrum of **6/7** in CD_3OD.

Figure S36. HMBC spectrum of **6/7** in CD_3OD.

Figure S37. NOESY spectrum of **6/7** in CD_3OD.

Figure S38. HRESIMS spectrum of **6/7** in CD_3OD.

Figure S39. Chiral-phase HPLC analyses of compounds **6** and **7**.

Original Data of Antibacterial Activity Assay

Compounds **1–28** were test for antibacterial activities against *Staphylococcus aureus* (ATCC 29213) using the agar filter paper diffusion [1]. *S. aureus* stored in glycerinum were activated in LB medium (10 g tryptone, 5 g yeast extract, 10 g NaCl, distilled water added up to 1,000 mL, pH 7.2–7.4) in a shaker-incubator at 37 °C and 180 r.p.m for 24 h. Then dilution-plate method was used to get effective concentration of 10^6–10^7 CFU ml^{-1}. The plate of LB medium was painted with 100 μL of *S. aureus* with effective concentration. The sterile filter paper (a diameter of 5 mm) was painted with each 10 μL of DMSO as negative control, ampicillin (0.5 mg/mL) as positive control and compounds **1–28** (5 mg/mL), respectively. Compounds **10** and **25** with 50 μg/disc displayed an inhibition zone with a diameter of about 21 and 15 mm, and ampicillin with 5 μg/disc showed an inhibition zone with a diameter of about 30 mm (Figure S1), respectively. Further, their minimum inhibitory concentrations (MIC) were evaluated in 96-well microtiter plates using a modification of the broth microdilution method [1]. Each well was added 100 μL of *S. aureus* with effective concentration, 90 μL of sterile LB medium and 10 μL of tested compounds, respectively. Compounds **10**,**25** and ampicillin with different concentration were added with final concentration as showed in Table S1 and replicated three times. The MIC value of compound **25** was 31.25 μg/mL, while compound **10** showed more than 500 μg/mL. Ampicillin was used as positive control with the MIC value of 6.25 μg/mL.

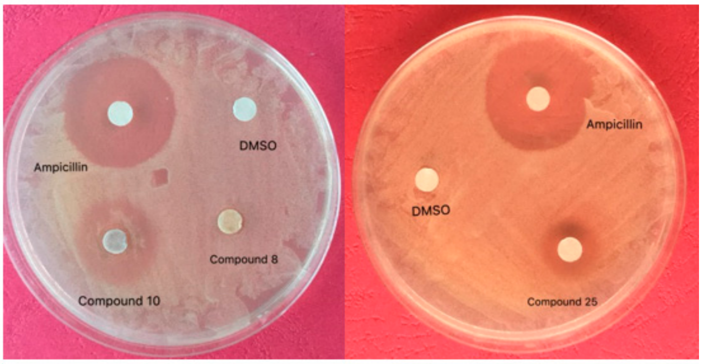

Figure S1 Pictures of inhibition zones in the filter paper diffusion test.

Table S1 The growth conditions of the *Staphylococcus aureus* after add different dilution concentration of compounds **10**, **25** and ampicillin.

Concentration μg/mL	500	250	125	62.5	31.25	15.63	7.82	3.91	1.96	0.98	0.49	DMSO
10	+	+	+	+	+	+	+	+	+	+	+	+
25	–	–	–	–	–	+	+	+	+	+	+	+
Concentration μg/mL	25	12.5	6.25	3.13	1.57	0.79	0.40	0.20	0.10	0.05	0.03	DMSO
Ampicillin	–	–	–	+	+	+	+	+	+	+	+	+

"+" indicated some *S. aureus* had grown. "–" indicated no *S. aureus* grew.

Detailed Process of Antitumor Activity Assay

Cytotoxicity was assayed with the CCK-8 (Dojindo, Kumamoto Prefecture, Japan) method [2]. Cell lines K562, SGC-7901 and BEL-7402 were purchased from Shanghai Cell Bank, Chinese Academy of Sciences. Cells were routinely grown and maintained in DMEM or RPMI media with 10% fetal bovine serum and with 1% streptomycin/penicillin. All cell lines were incubated in a Thermo/ Forma Scientific CO_2 water-jacketed incubator with 5% CO_2 in air at 37 °C. A cell viability assay was

determined with the CCK-8 assay. Cells were seeded at a density of 400–800 cells/well in 384-well plates and treated with various concentrations of compounds or solvent control. After 72 h incubation, CCK-8 reagent was added, and absorbance was measured at 450 nm using an Envision 2104 multilabel reader (PerkinElmer, Waltham, MA, USA). Dose−response curves were plotted to determine the IC_{50} values using Prism 5.0 (GraphPad Software Inc., San Diego, CA, USA). Taxol was used as the positive control, with IC_{50} values of 0.18 ± 0.20, 0.89 ± 0.15 and 0.54 ± 0.20 μg/mL, respectively.

The 16S rRNA Gene Sequences Data of *Alternaria* sp. SCSIO41014

CTGGATCTCTCGGGGTTACAGCCTTGCTGAATTATTCACCCTTGTCTTTTGCGTACTTCTTGTT
TCCTTGGTGGGTTCGCCCACCACTAGGACAAACATAAACCTTTTGTAATTGCAATCAGCGTC
AGTAACAAATTAATAATTACAACTTTCAACAACGGATCTCTTGGTTCTGGCATCGATGAAG
AACGCAGCGAAATGCGATAAGTAGTGTGAATTGCAGAATTCAGTGAATCATCGAATCTTTG
AACGCACATTGCGCCCTTTGGTATTCCAAAGGGCATGCCTGTTCGAGCGTCATTTGTACCCT
CAAGCTTTGCTTGGTGTTGGGCGTCTTGTCTCTAGCTTTGCTGGAGACTCGCCTTAAAGTAAT
TGGCAGCCGGCCTACTGGTTTCGGAGCGCAGCACAAGTCGCACTCTCTATCAGCAAAGGTC
TAGCATCCATTAAGCCTTTTTTCAACTTTTGACCTCGGATCAGGTAGGGATACCCGCTGAAC
TTAAGCATATCA

Theory and Calculation Details of Compound 1

To determine the absolute configuration of **1**, a computational modeling study was conducted using the Gaussian 03 program package [3,4]. The ECD of the lowest energy conformer was then calculated by the TDDFT method at the B3LYP/6-31G(d) level in methanol solution.

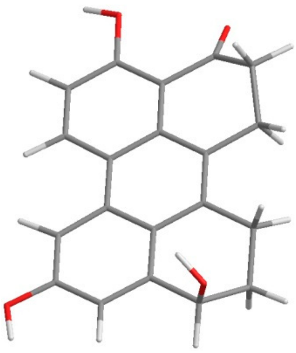

B3LYP/6-31G(d) optimized lowest energy 3D conformer of **1**.

References

1. Wang, J.F.; Cong, Z.W.; Huang, X.L.; Hou, C.X.; Chen, W.B.; Tu, Z.C.; Huang, D.Y.; Liu, Y.H. *Org. Lett.* **2018**, *20*, 1371–1374.
2. Wang, J.F.; Wei, X.Y.; Qin, X.C.; Tian, X.P.; Liao, L.; Li, K.M.; Zhou, X.F.; Yang, X.W.; Wang, F.Z.; Zhang, T.Y.; et al. J. Nat. Prod. 2016, *79*, 59–65.
3. Gaussian 03, Revision E.01, Frisch, M.J.; Trucks, G.W.; Schlegel, H.B.; Scuseria, G.E.; Robb, M.A.; Cheeseman, J.R.; Montgomery, J.A.; Vreven, Jr.T.; Kudin, K.N.; Burant, J.C.; et al.; Gaussian, Inc., Wallingford CT, 2004. Available online: http://gaussian.com/g03citation/ (accessed on 8 August 2018).
4. Sai, C.; Li, D.; Xue, C.; Wang, K.; Hu, P.; Pei, Y.; Bai, J.; Jing, Y.; Li, Z.; Hua, H. Org. Lett. **2015**, *17*, 4102–4105.

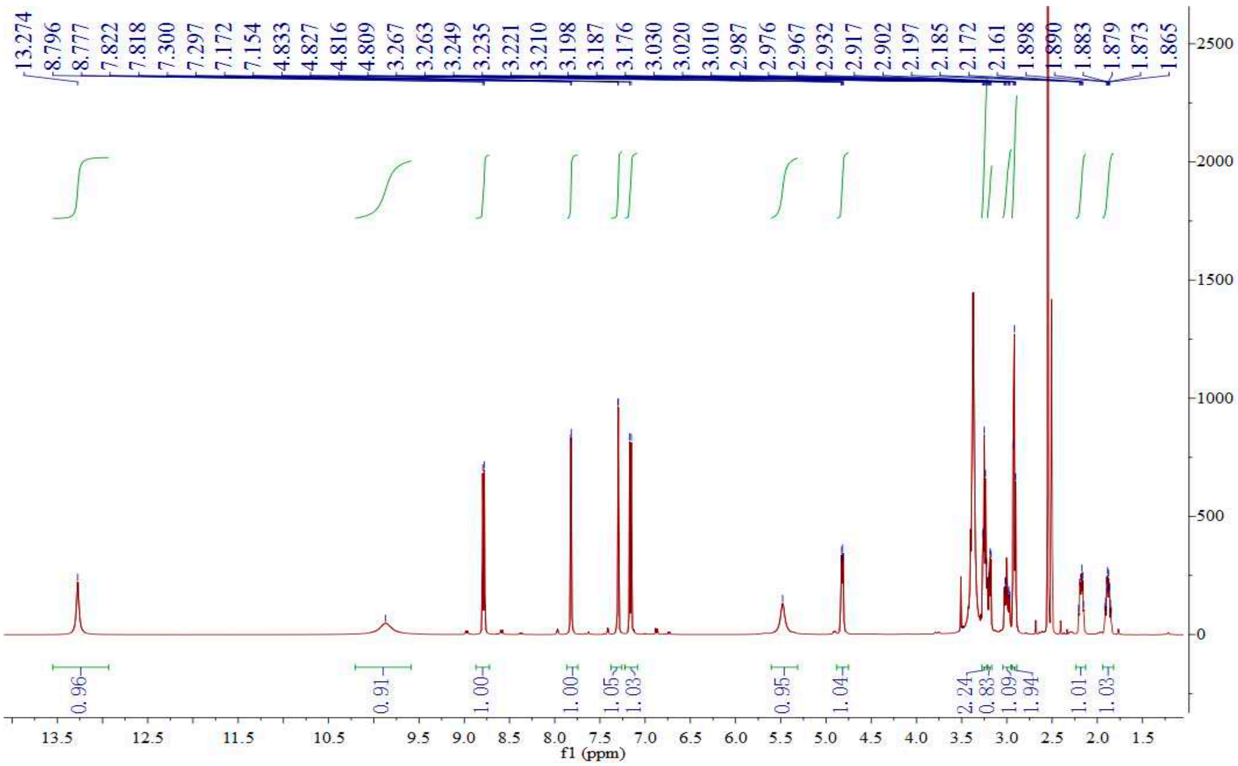

Figure S2. ^{1}H NMR spectrum of **1** in DMSO-d_6.

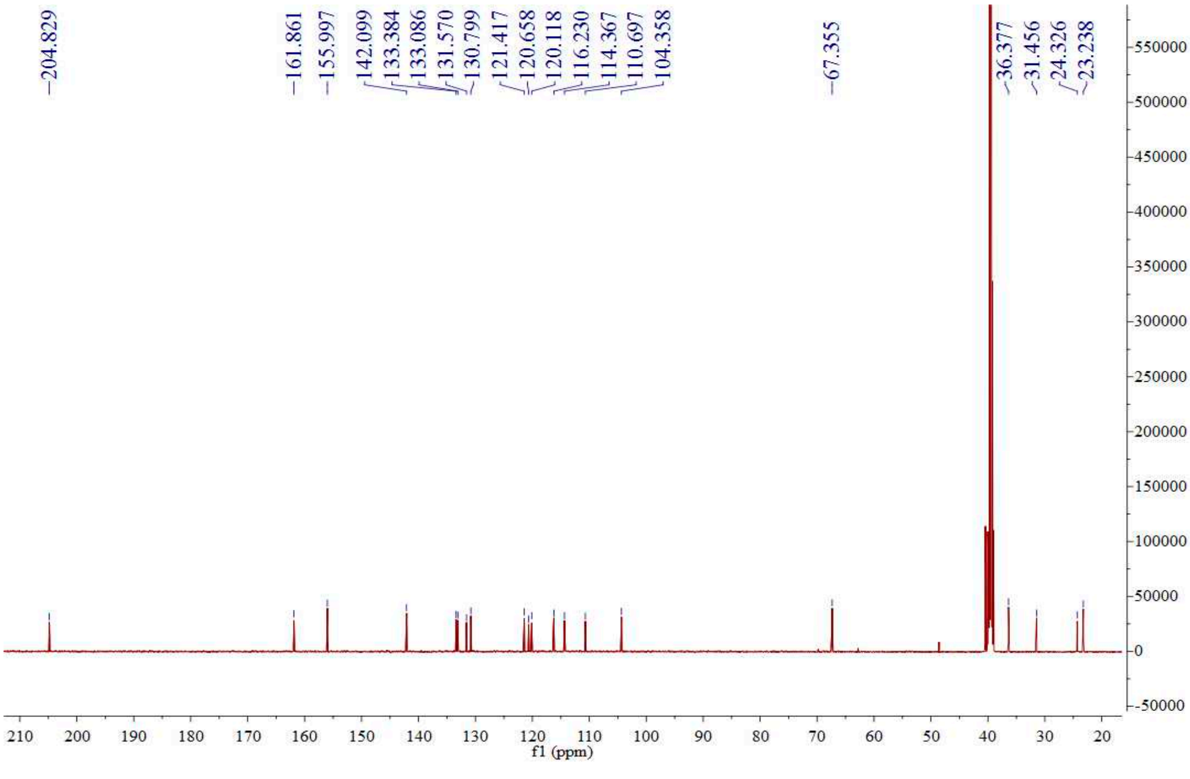

Figure S3. ^{13}C NMR spectrum of **1** in DMSO-d_6.

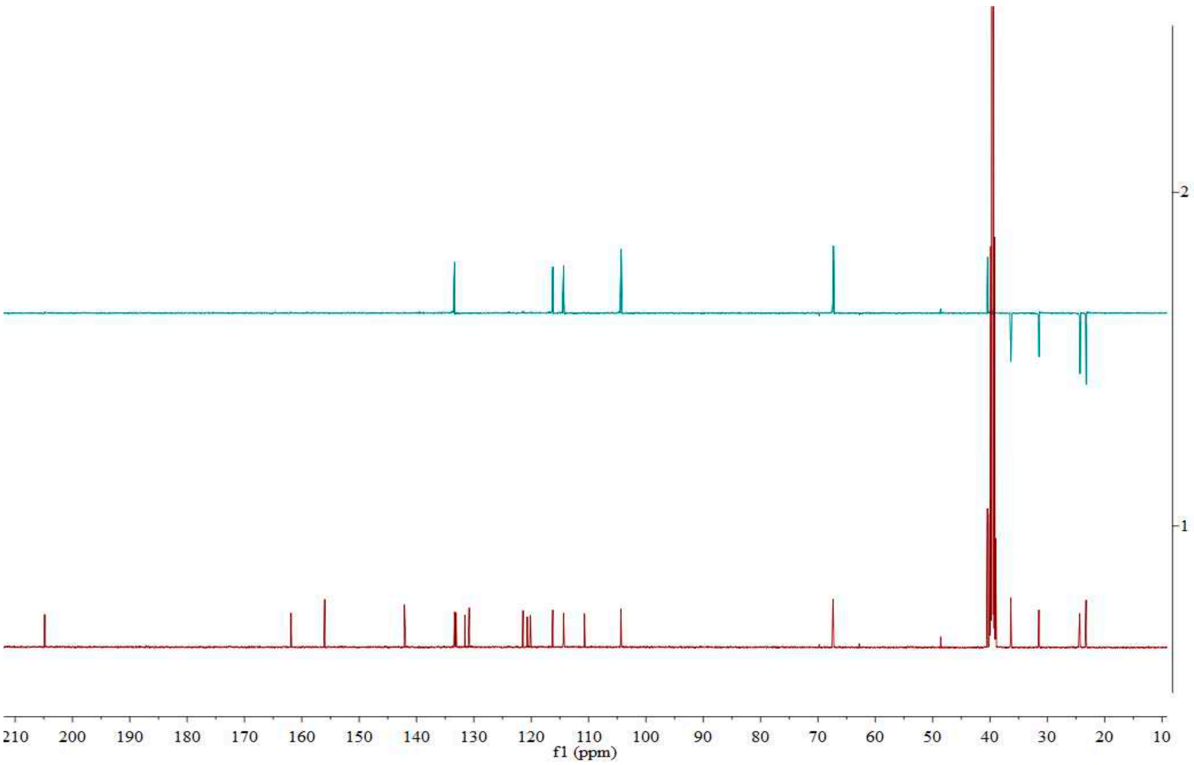

Figure S4. DEPT NMR spectrum of **1** in DMSO-*d*6.

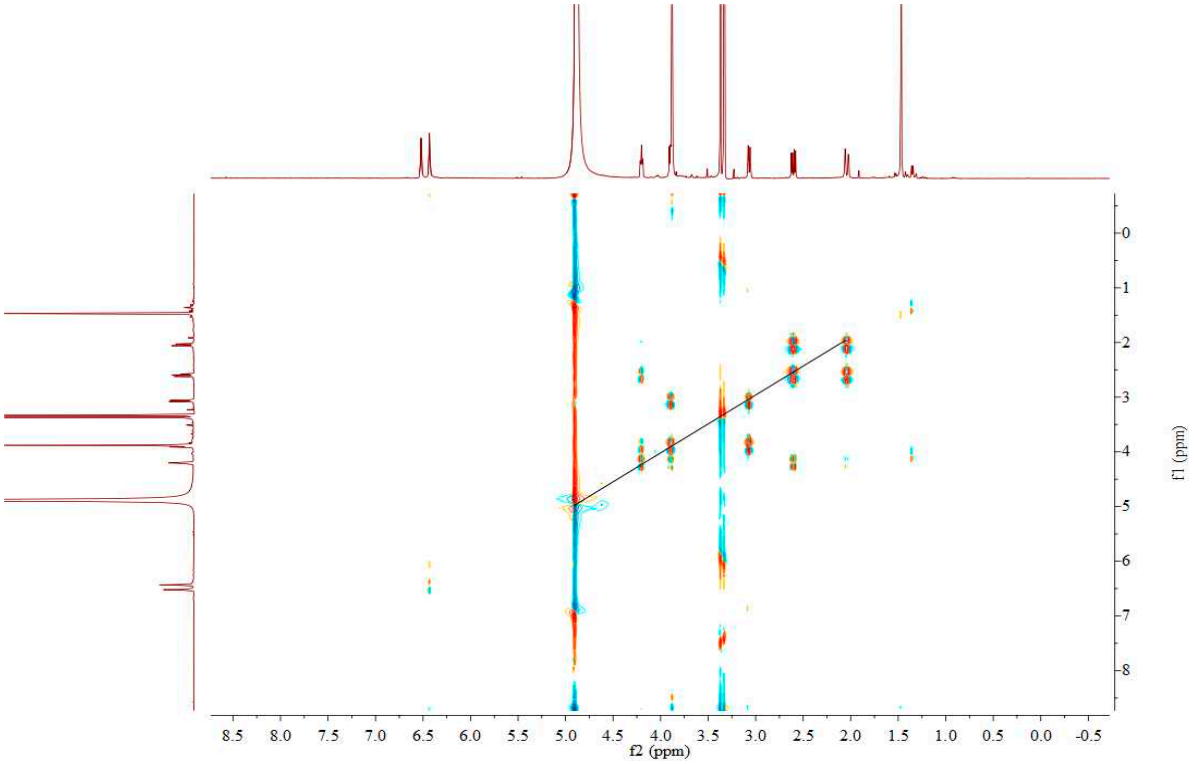

Figure S5. ^{1}H-^{1}H COSY spectrum of **1** in DMSO-*d*6.

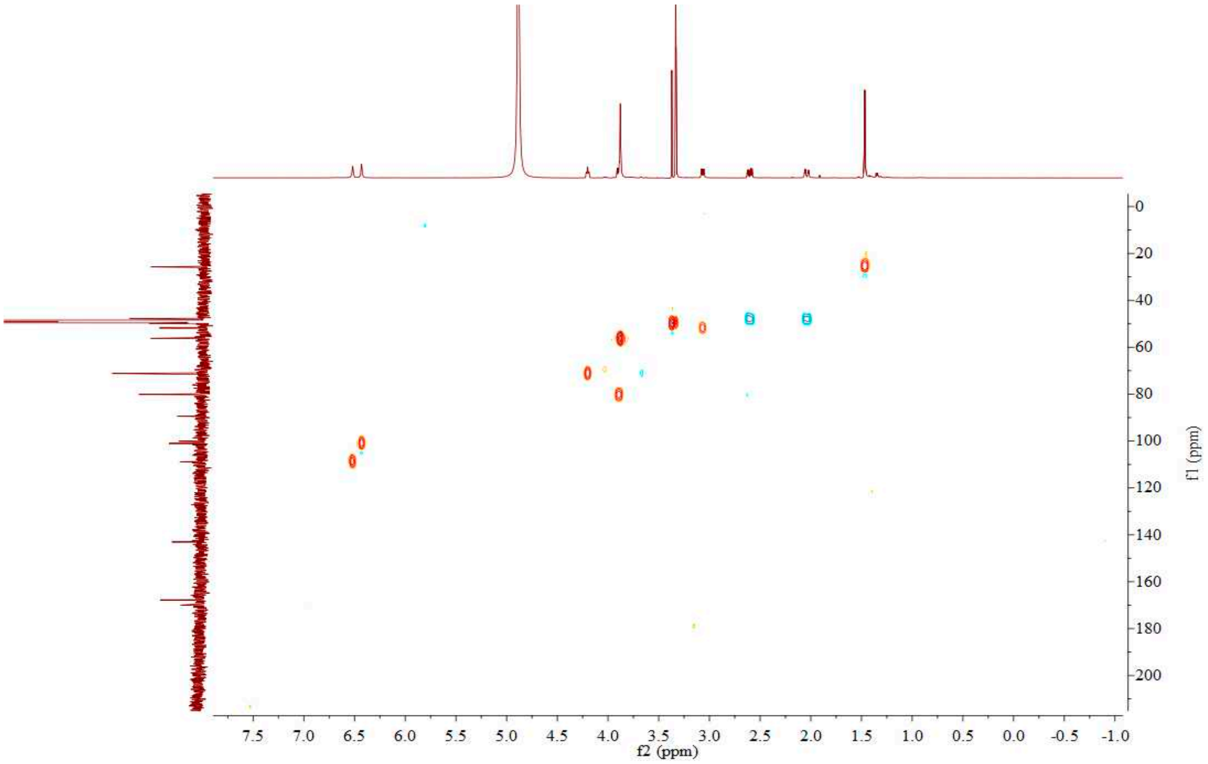

Figure S6. HSQC spectrum of **1** in DMSO-*d*6.

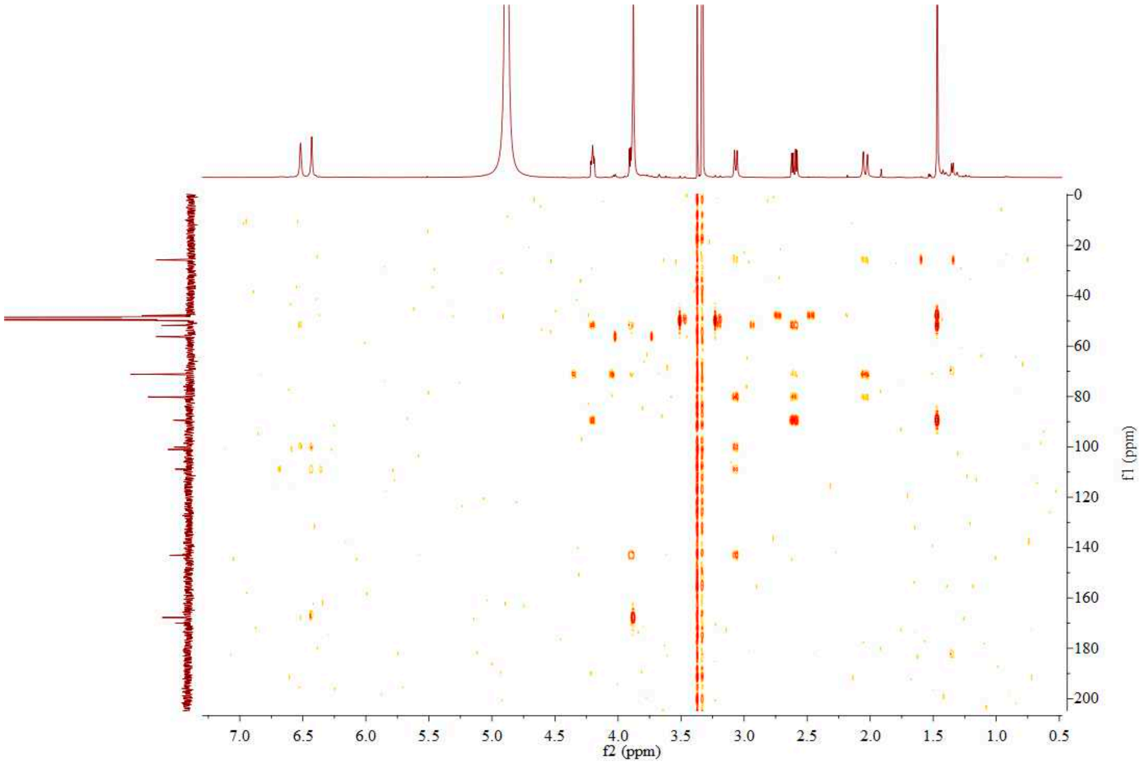

Figure S7. HMBC spectrum of **1** in DMSO-*d*6.

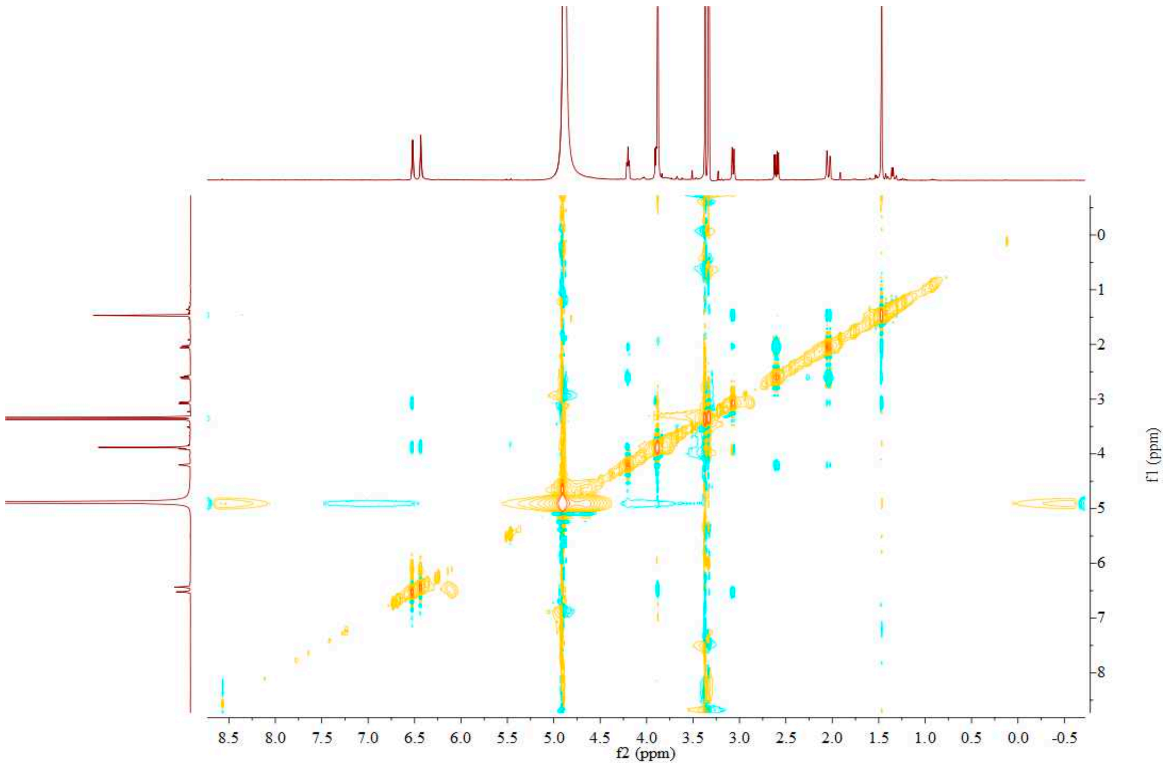

Figure S8. NOESY spectrum of **1** in DMSO-d_6.

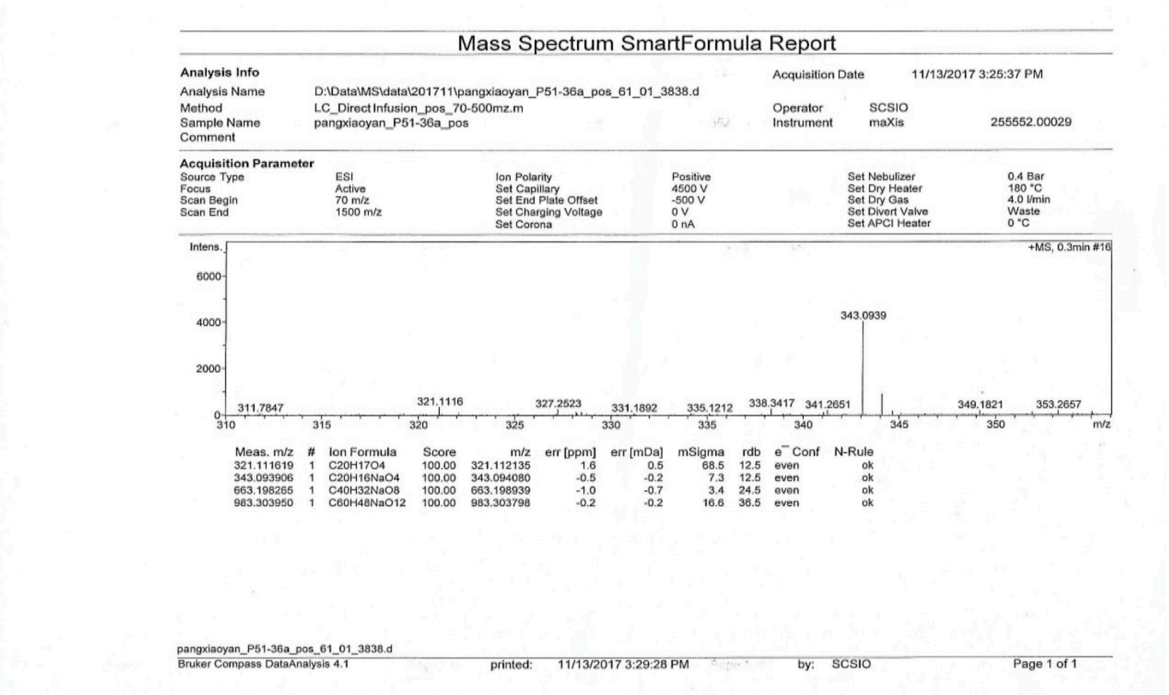

Mass Spectrum SmartFormula Report

Analysis Info

Analysis Name D:\Data\MS\data\201711\pangxiaoyan_P51-36a_pos_61_01_3838.d
Method LC_Direct Infusion_pos_70-500mz.m
Sample Name pangxiaoyan_P51-36a_pos
Comment

Acquisition Date 11/13/2017 3:25:37 PM
Operator SCSIO
Instrument maXis 255552.00029

Acquisition Parameter

Source Type	ESI	Ion Polarity	Positive	Set Nebulizer	0.4 Bar
Focus	Active	Set Capillary	4500 V	Set Dry Heater	180 °C
Scan Begin	70 m/z	Set End Plate Offset	-500 V	Set Dry Gas	4.0 l/min
Scan End	1500 m/z	Set Charging Voltage	0 V	Set Divert Valve	Waste
		Set Corona	0 nA	Set APCI Heater	0 °C

Meas. m/z	#	Ion Formula	Score	m/z	err [ppm]	err [mDa]	mSigma	rdb	e⁻ Conf	N-Rule
321.111619	1	C20H17O4	100.00	321.112135	1.6	0.5	68.5	12.5	even	ok
343.093906	1	C20H16NaO4	100.00	343.094080	-0.5	-0.2	7.3	12.5	even	ok
663.198265	1	C40H32NaO8	100.00	663.198939	-1.0	-0.7	3.4	24.5	even	ok
983.303950	1	C60H48NaO12	100.00	983.303798	-0.2	-0.2	16.6	36.5	even	ok

pangxiaoyan_P51-36a_pos_61_01_3838.d
Bruker Compass DataAnalysis 4.1 printed: 11/13/2017 3:29:28 PM by: SCSIO Page 1 of 1

Figure S9. HRESIMS spectrum of **1** in CD_3OD.

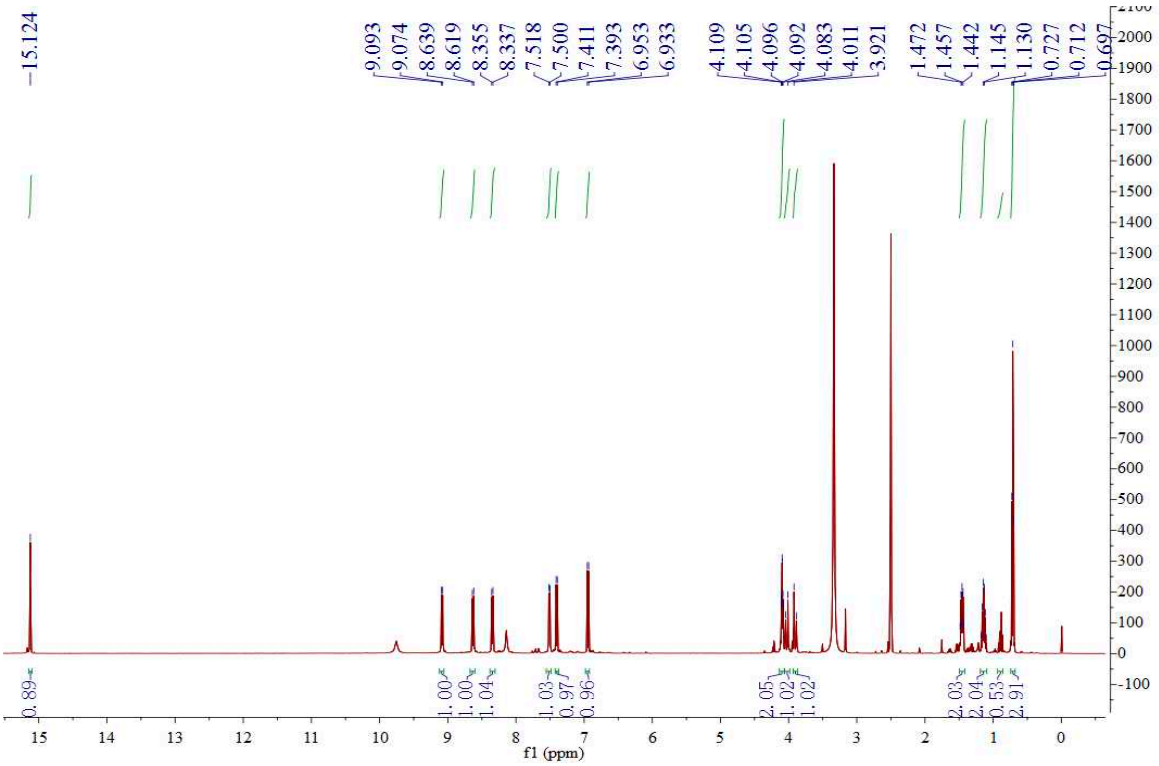

Figure S10. ^{1}H NMR spectrum of **2** in DMSO-d_6.

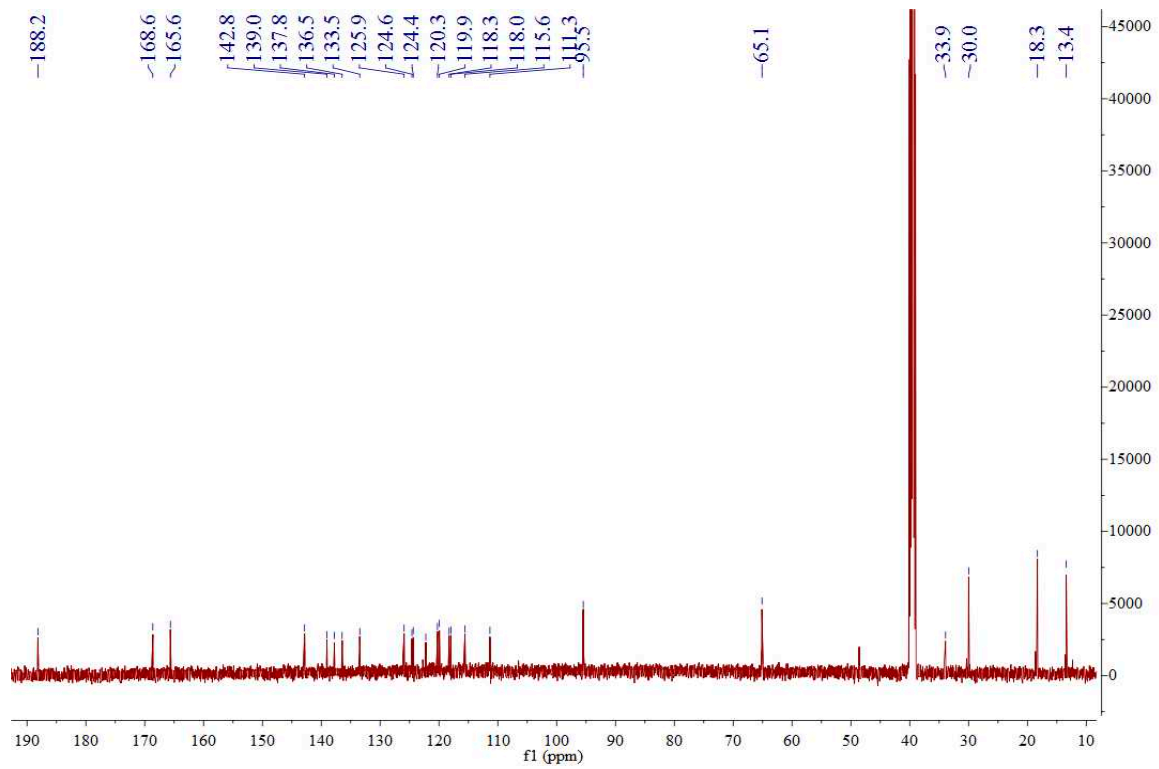

Figure S11. ^{13}C NMR spectrum of **2** in DMSO-d_6.

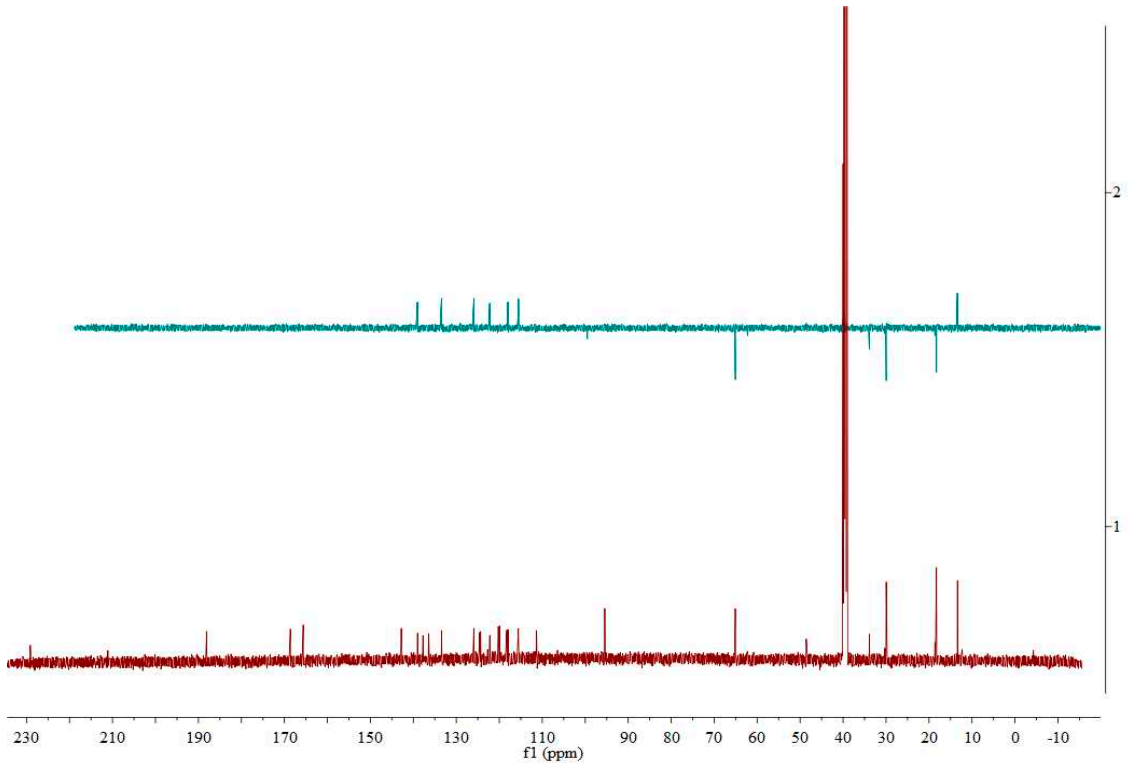

Figure S12. DEPT NMR spectrum of **2** in DMSO-d_6.

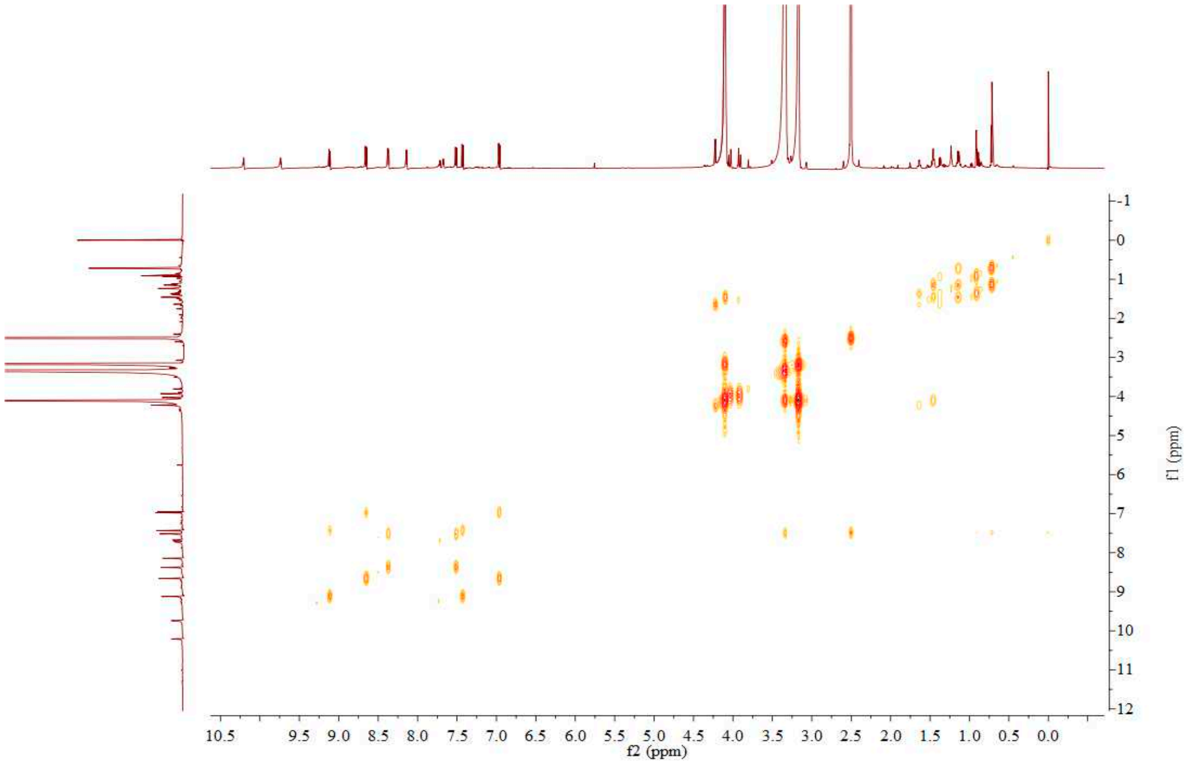

Figure S13. ^{1}H-^{1}H COSY spectrum of **2** in DMSO-d_6.

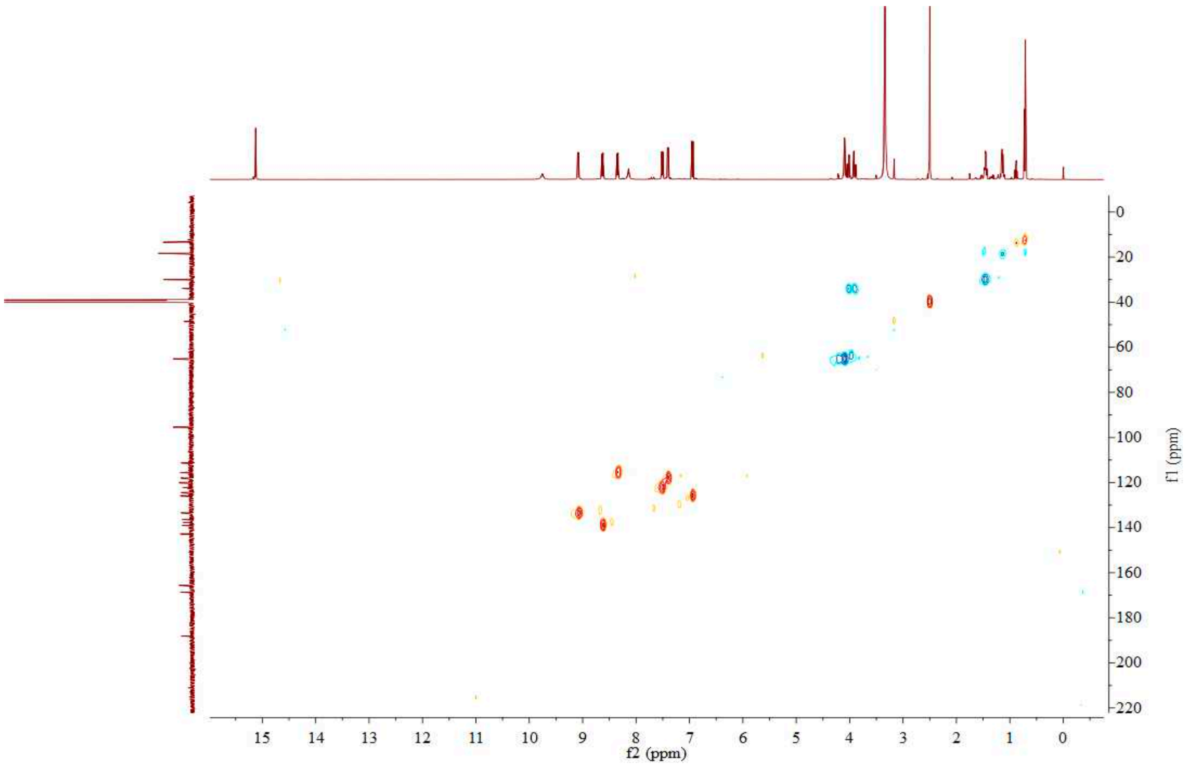

Figure S14. HSQC spectrum of **2** in DMSO-d_6.

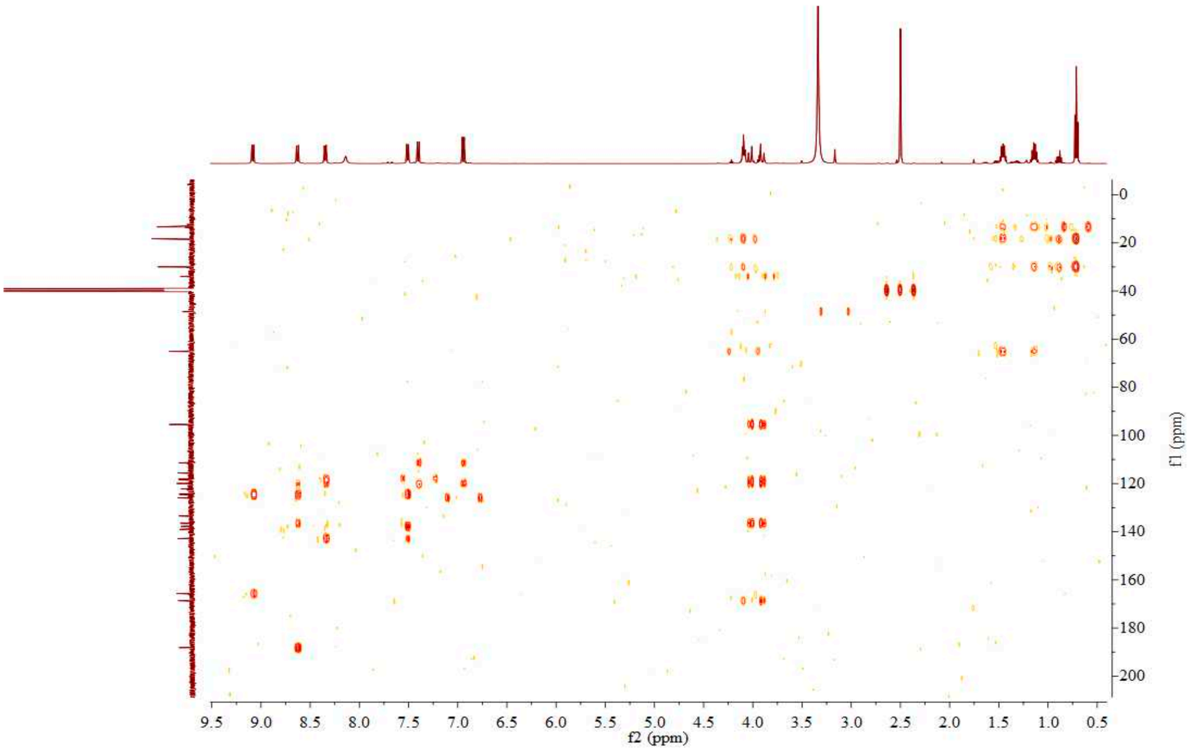

Figure S15. HMBC spectrum of **2** in DMSO-d_6.

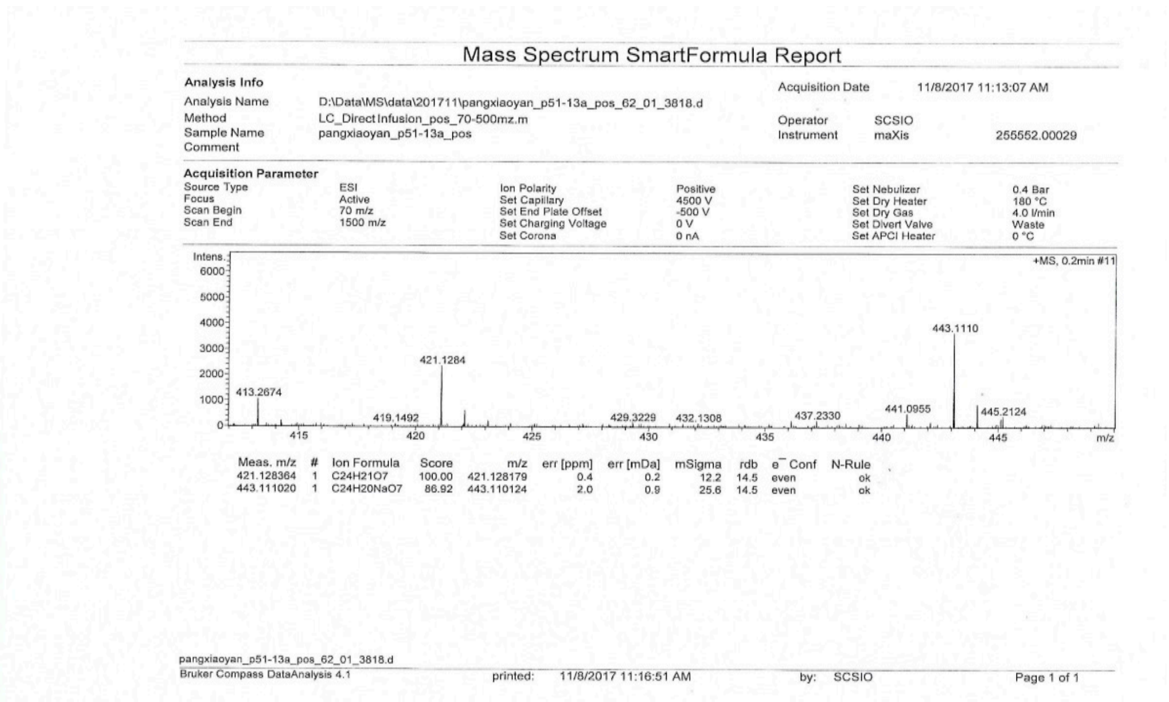

Figure S16. HRESIMS spectrum of **2** in CD_3OD.

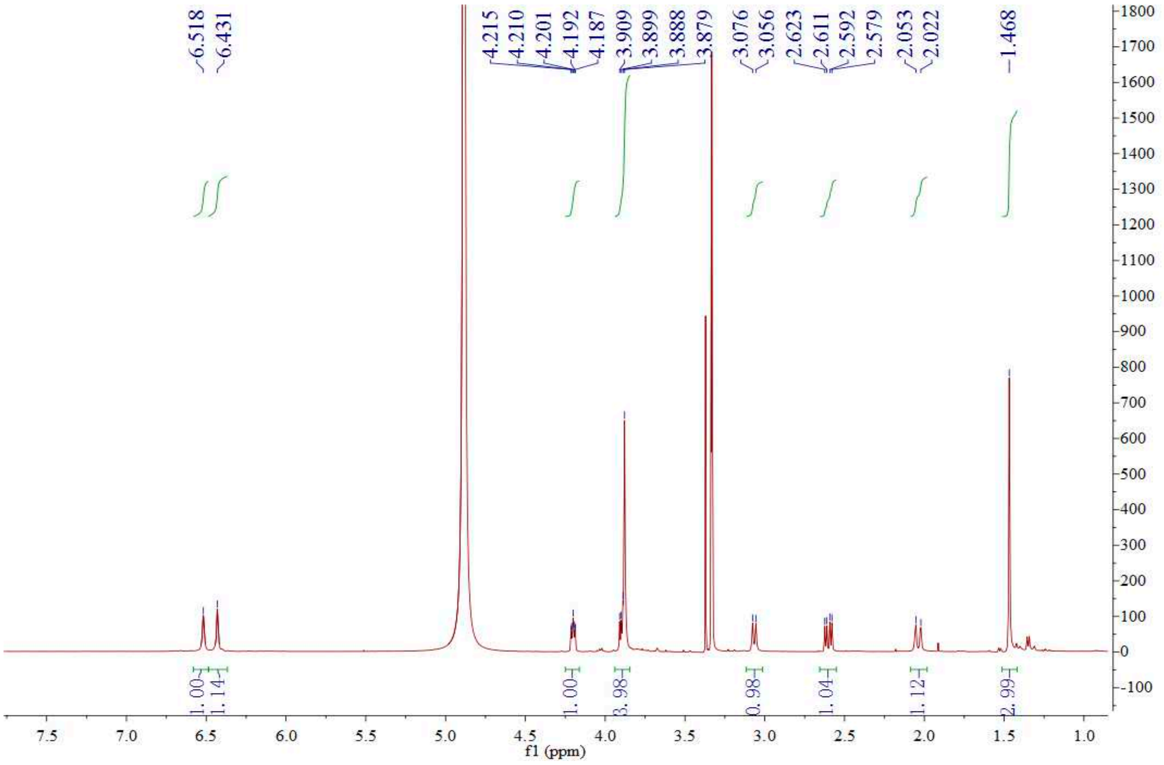

Figure S17. ^{1}H NMR spectrum of **3** in CD_3OD.

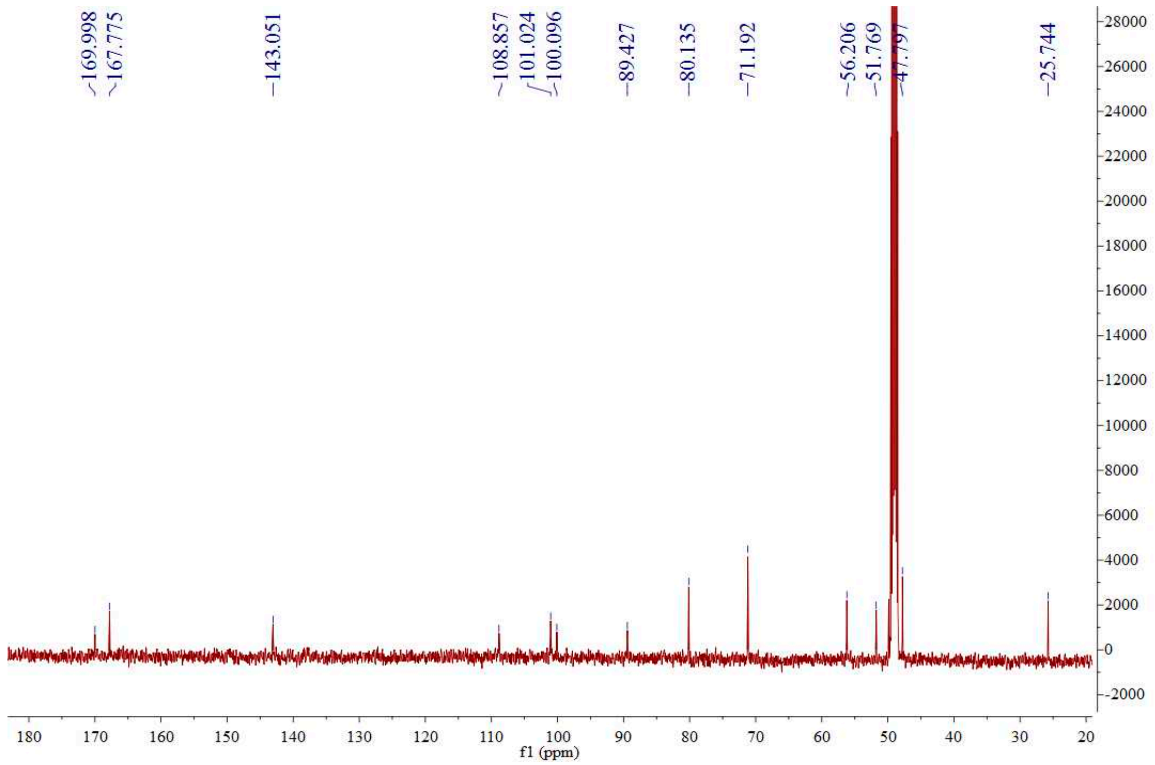

Figure S18. ^{13}C NMR spectrum of **3** in CD_3OD.

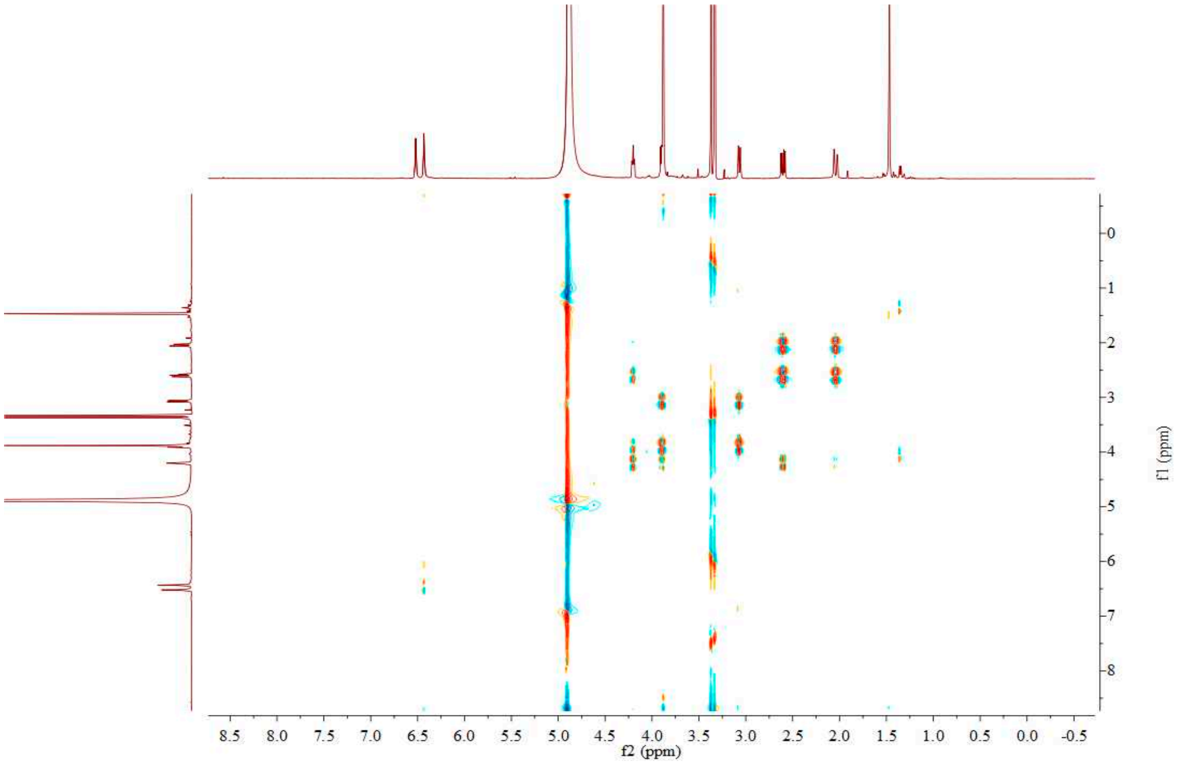

Figure S19. ^1H-^1H COSY spectrum of **3** in CD_3OD.

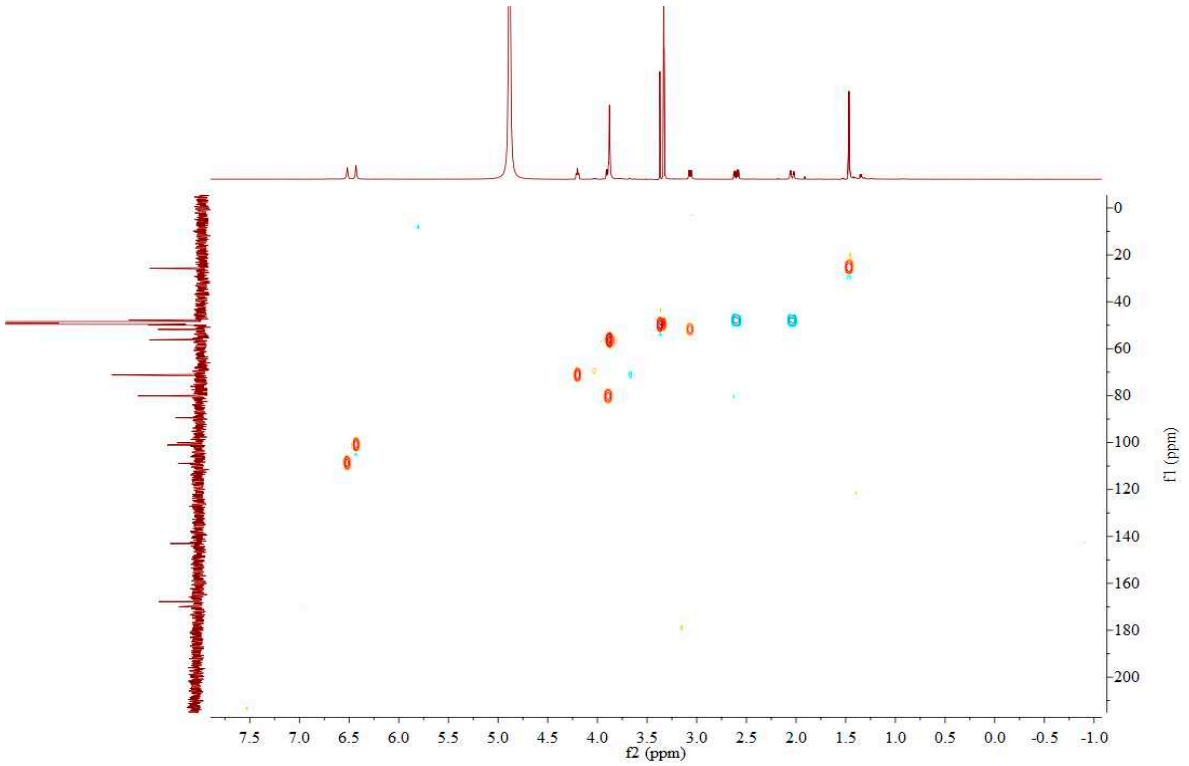

Figure S20. HSQC spectrum of **3** in CD_3OD.

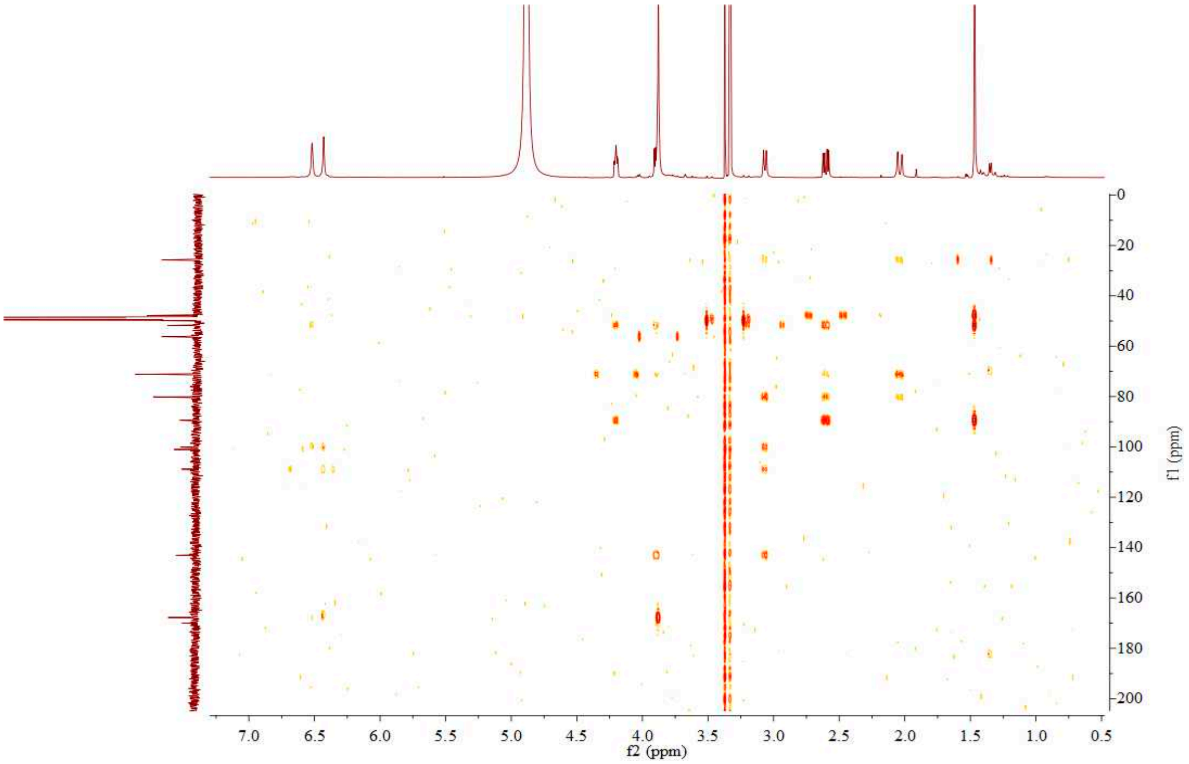

Figure S21. HMBC spectrum of **3** in CD_3OD.

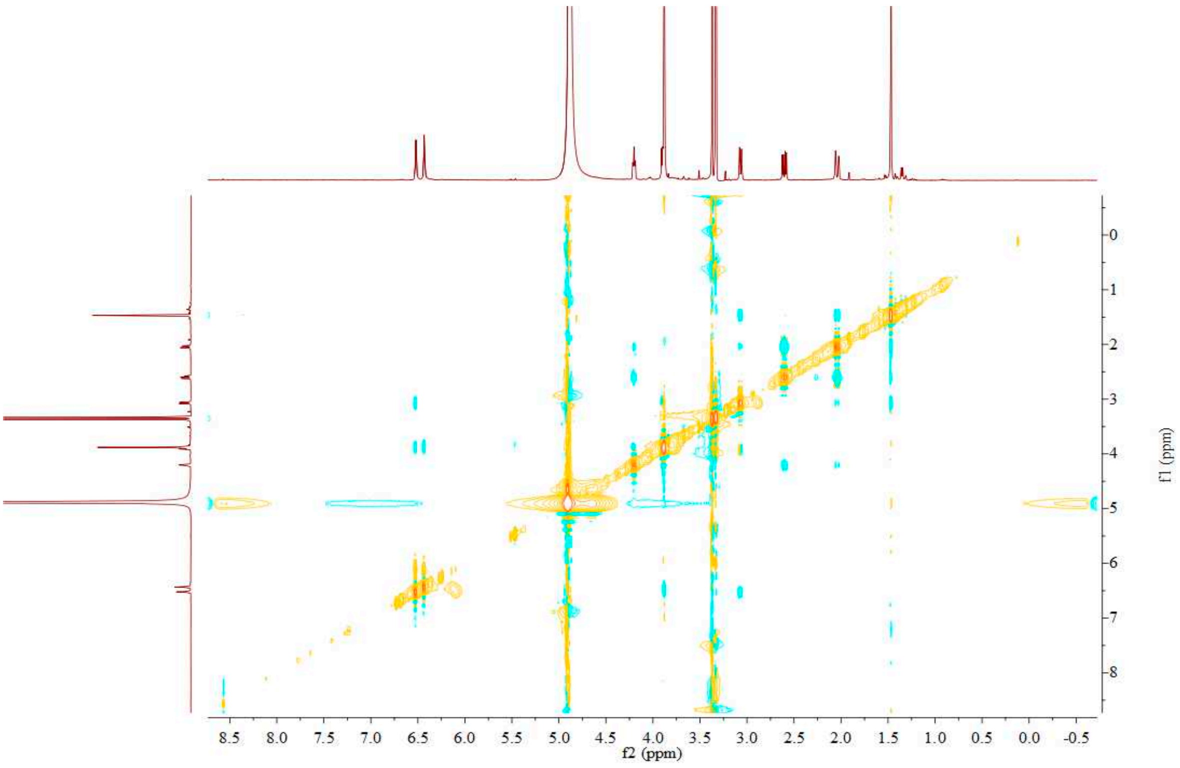

Figure S22. NOESY spectrum of **2** in CD3OD.

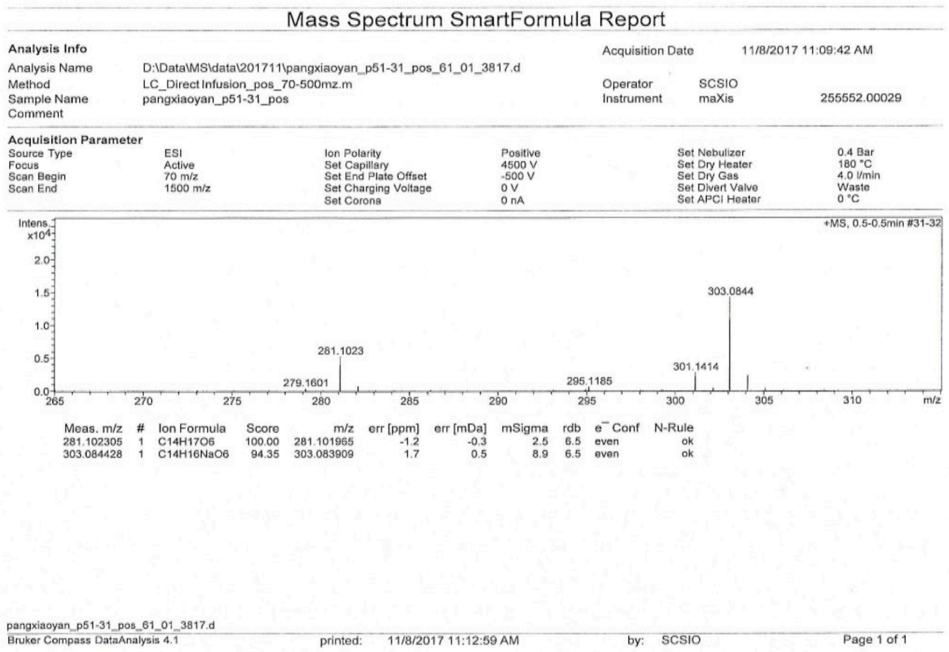

Mass Spectrum SmartFormula Report

Analysis Info
Analysis Name D:\Data\MS\data\201711\pangxiaoyan_p51-31_pos_61_01_3817.d
Method LC_Direct Infusion_pos_70-500mz.m
Sample Name pangxiaoyan_p51-31_pos
Comment
Acquisition Date 11/8/2017 11:09:42 AM
Operator SCSIO
Instrument maXis 255552.00029

Acquisition Parameter

Source Type	ESI	Ion Polarity	Positive	Set Nebulizer	0.4 Bar
Focus	Active	Set Capillary	4500 V	Set Dry Heater	180 °C
Scan Begin	70 m/z	Set End Plate Offset	-500 V	Set Dry Gas	4.0 l/min
Scan End	1500 m/z	Set Charging Voltage	0 V	Set Divert Valve	Waste
		Set Corona	0 nA	Set APCI Heater	0 °C

Meas. m/z	#	Ion Formula	Score	m/z	err [ppm]	err [mDa]	mSigma	rdb	e⁻ Conf	N-Rule
281.102305	1	C14H17O6	100.00	281.101965	-1.2	-0.3	2.5	6.5	even	ok
303.084428	1	C14H16NaO6	94.35	303.083909	1.7	0.5	8.9	6.5	even	ok

pangxiaoyan_p51-31_pos_61_01_3817.d
Bruker Compass DataAnalysis 4.1 printed: 11/8/2017 11:12:59 AM by: SCSIO Page 1 of 1

Figure S23. HRESIMS spectrum of **3** in CD3OD.

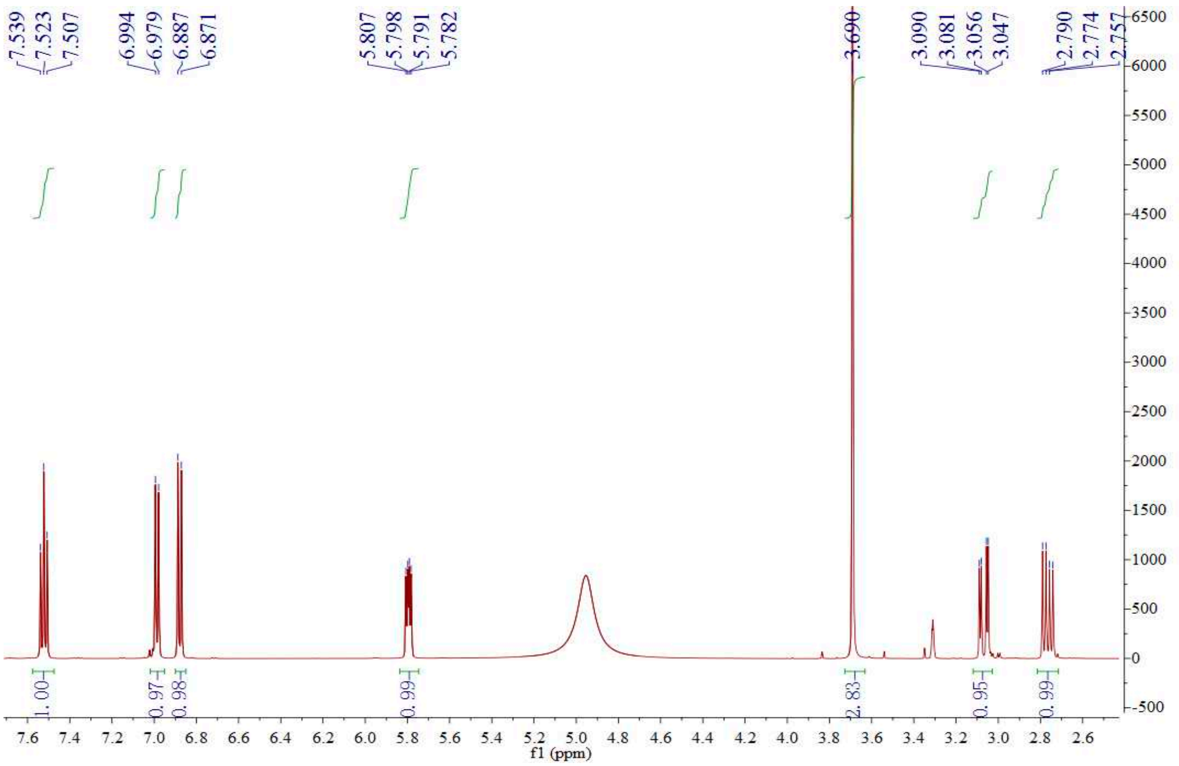

Figure S24. ^1H NMR spectrum of **4/5** in CD_3OD.

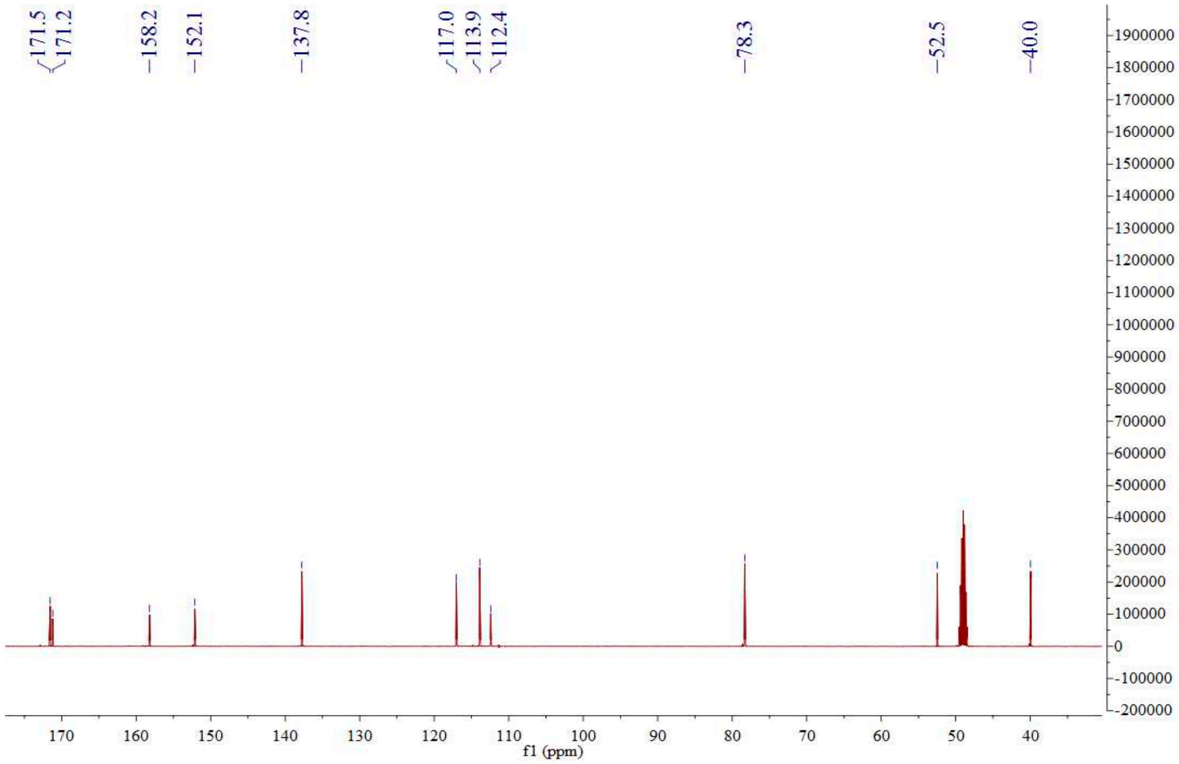

Figure S25. ^{13}C NMR spectrum of **4/5** in CD_3OD.

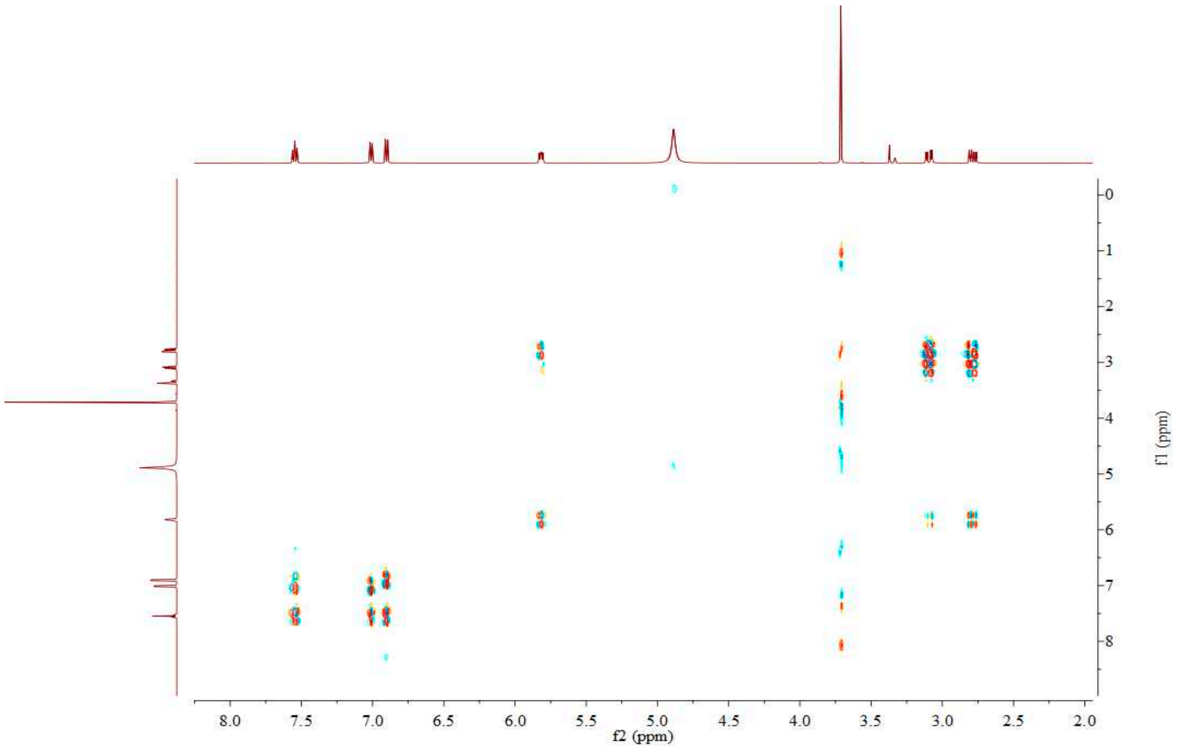

Figure S26. ^{1}H-^{1}H COSY spectrum of **4/5** in CD_3OD.

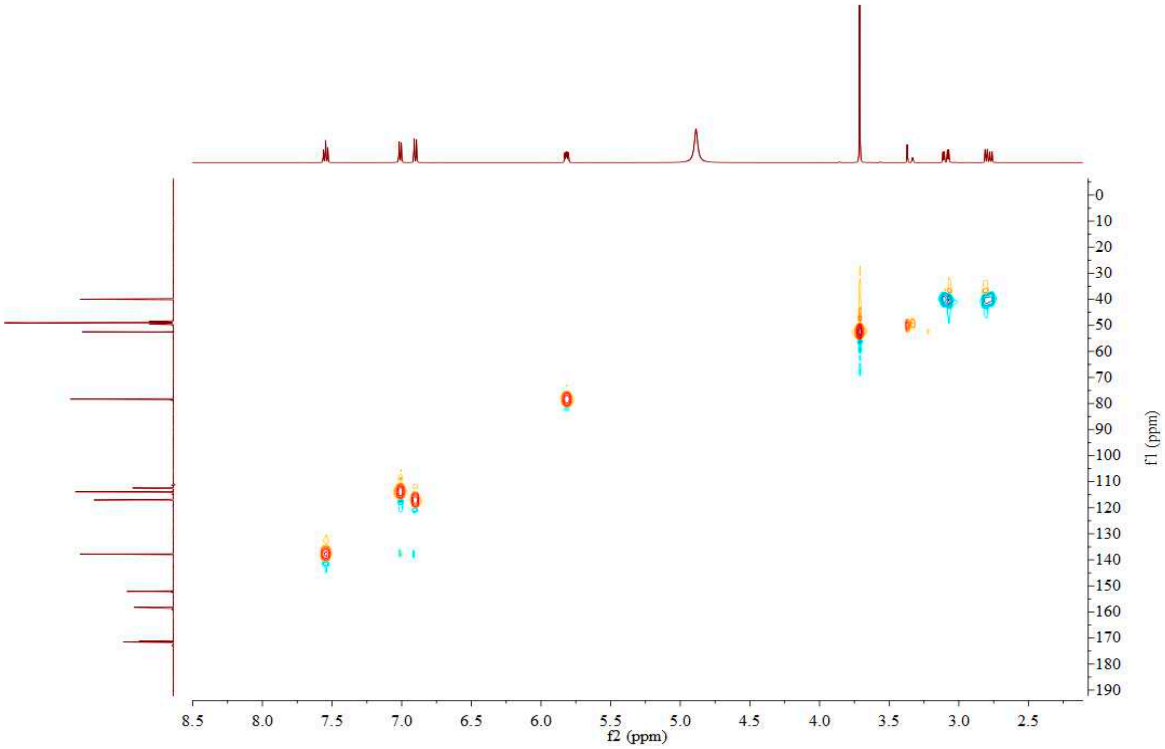

Figure S27. HSQC spectrum of **4/5** in CD_3OD.

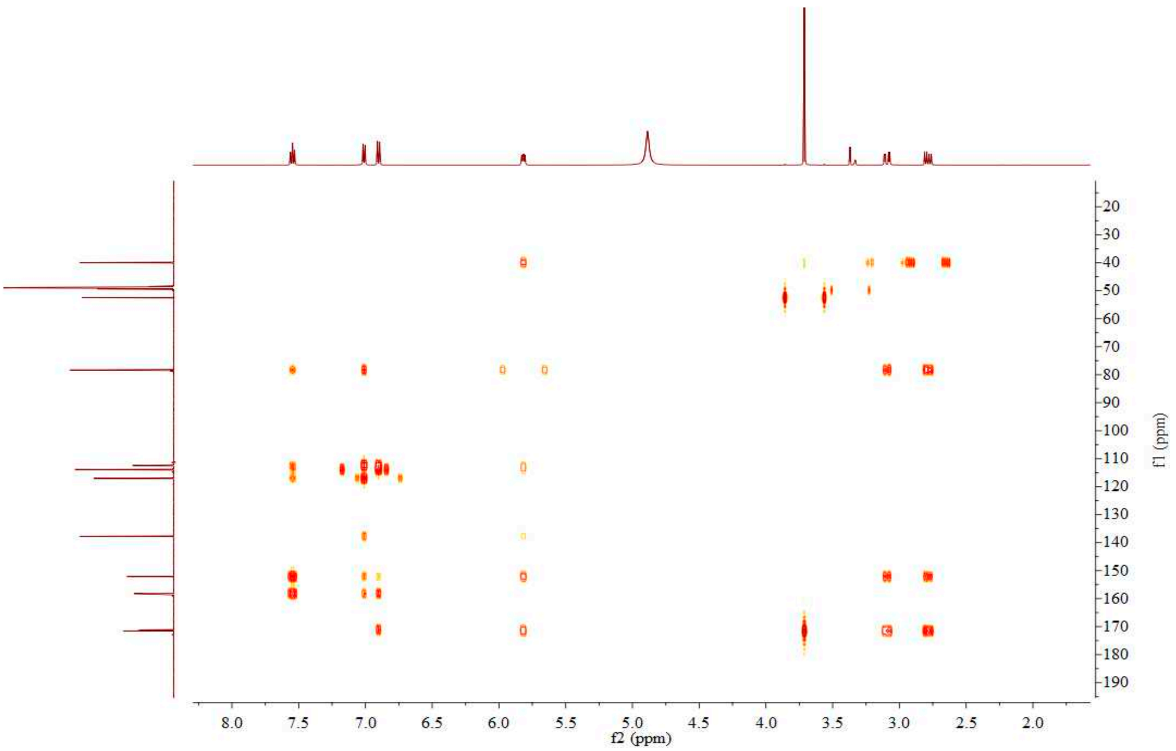

Figure S28. HMBC spectrum of **4/5** in CD3OD.

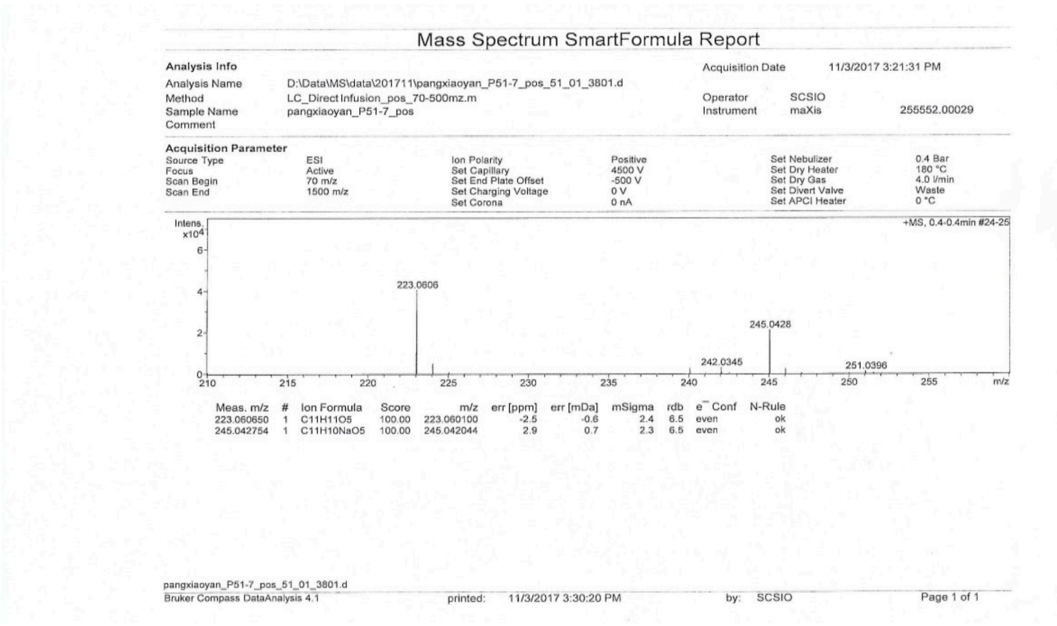

Mass Spectrum SmartFormula Report

Analysis Info
Analysis Name D:\Data\MS\data\201711\pangxiaoyan_P51-7_pos_51_01_3801.d
Method LC_Direct Infusion_pos_70-500mz.m
Sample Name pangxiaoyan_P51-7_pos
Comment
Acquisition Date 11/3/2017 3:21:31 PM
Operator SCSIO
Instrument maXis 255552.00029

Acquisition Parameter

Source Type	ESI	Ion Polarity	Positive	Set Nebulizer	0.4 Bar
Focus	Active	Set Capillary	4500 V	Set Dry Heater	180 °C
Scan Begin	70 m/z	Set End Plate Offset	-500 V	Set Dry Gas	4.0 l/min
Scan End	1500 m/z	Set Charging Voltage	0 V	Set Divert Valve	Waste
		Set Corona	0 nA	Set APCI Heater	0 °C

Meas. m/z	#	Ion Formula	Score	m/z	err [ppm]	err [mDa]	mSigma	rdb	e⁻ Conf	N-Rule
223.060650	1	C11H11O5	100.00	223.060100	-2.5	-0.6	2.4	6.5	even	ok
245.042754	1	C11H10NaO5	100.00	245.042044	2.9	0.7	2.3	6.5	even	ok

pangxiaoyan_P51-7_pos_51_01_3801.d
Bruker Compass DataAnalysis 4.1 printed: 11/3/2017 3:30:20 PM by: SCSIO Page 1 of 1

Figure S29. HRESIMS spectrum of **4/5** in CD3OD.

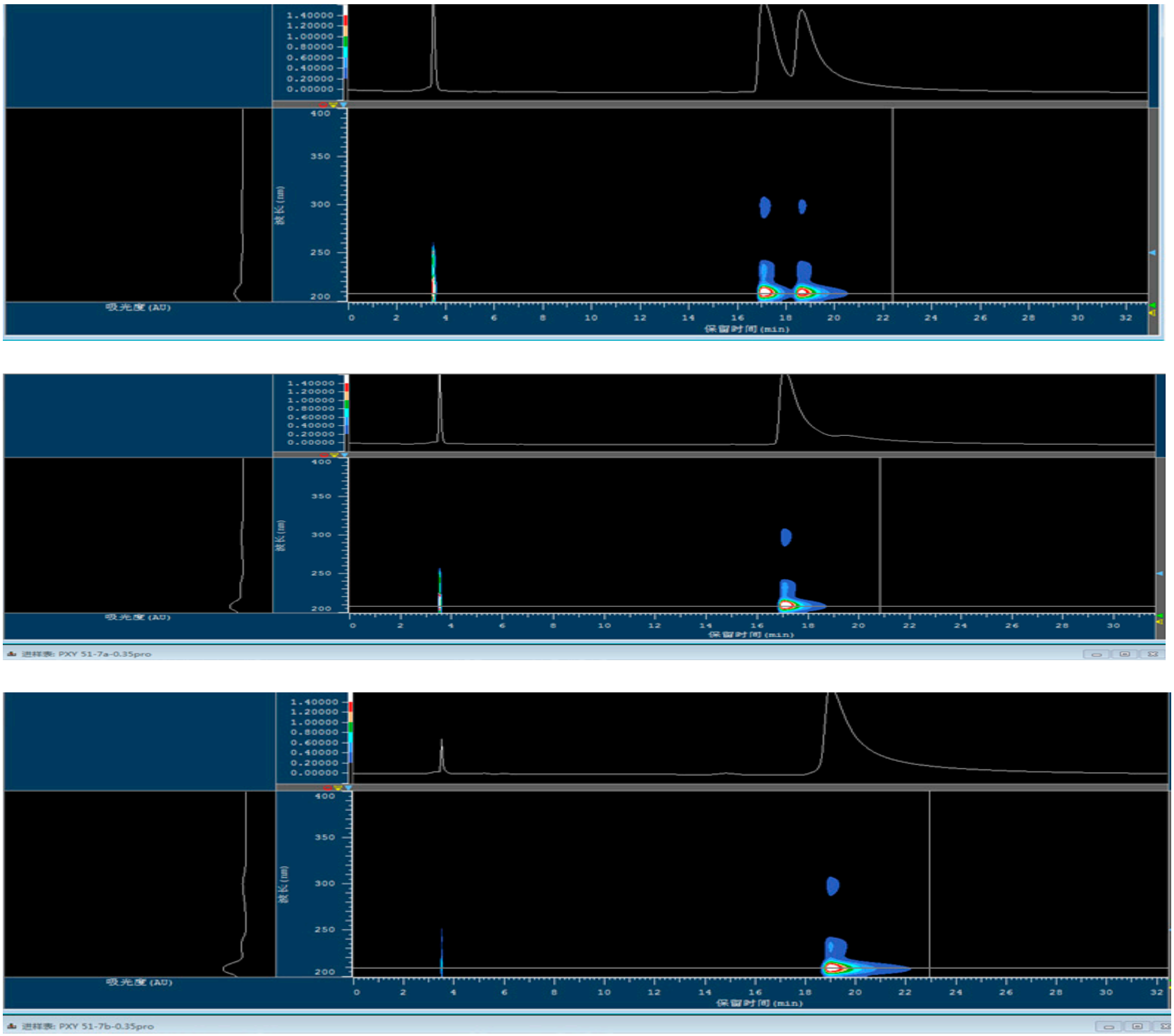

Figure S30. Chiral HPLC analyses of compounds **4** and **5** (Daicel Chiraloak IC-3 column, 4.6 mm × 25 mm, eluent n-hexane–iso-propanol, 65:35 *v*/*v*, 1 mL/min).

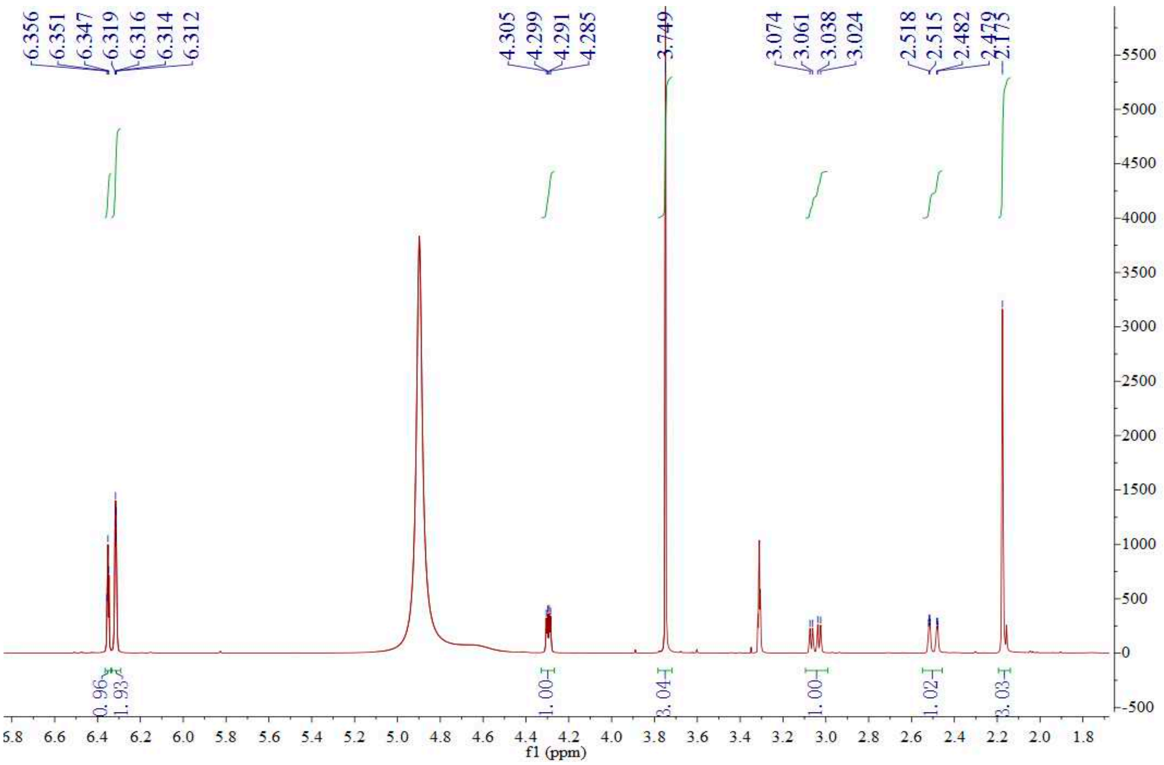

Figure S31. ^{1}H NMR spectrum of **6/7** in CD_3OD.

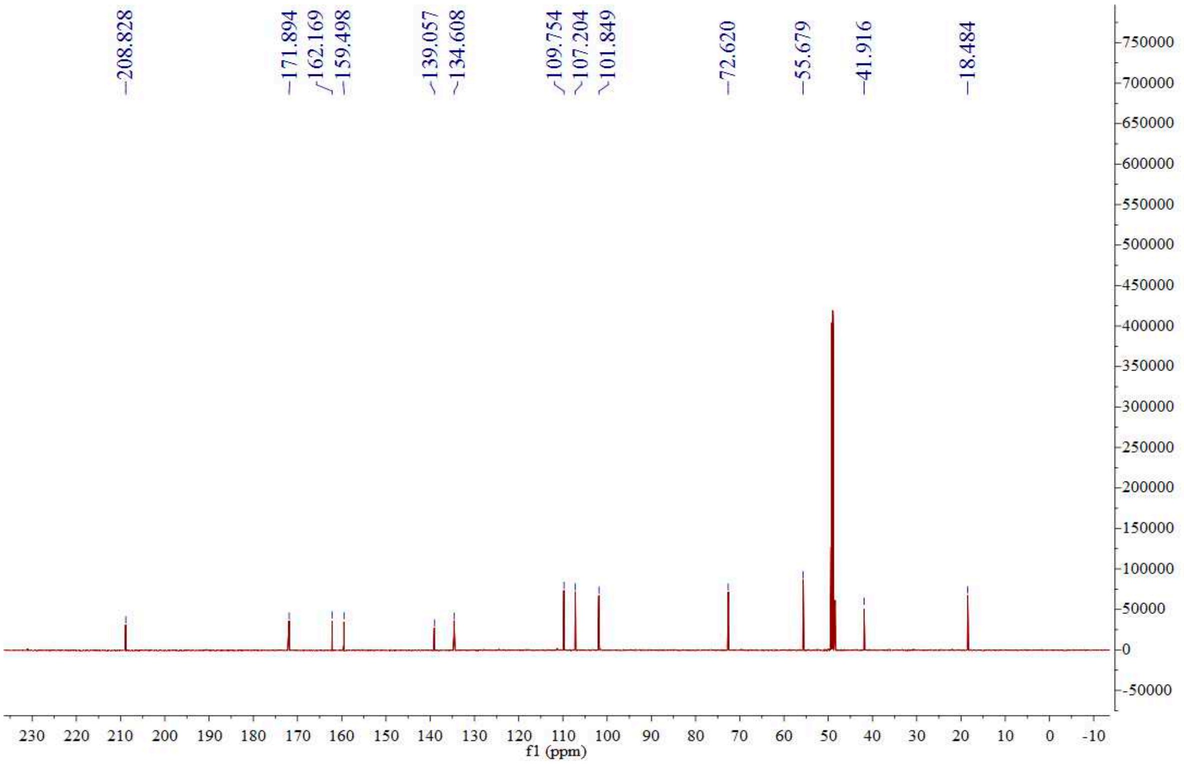

Figure S32. ^{13}C NMR spectrum of **6/7** in CD_3OD.

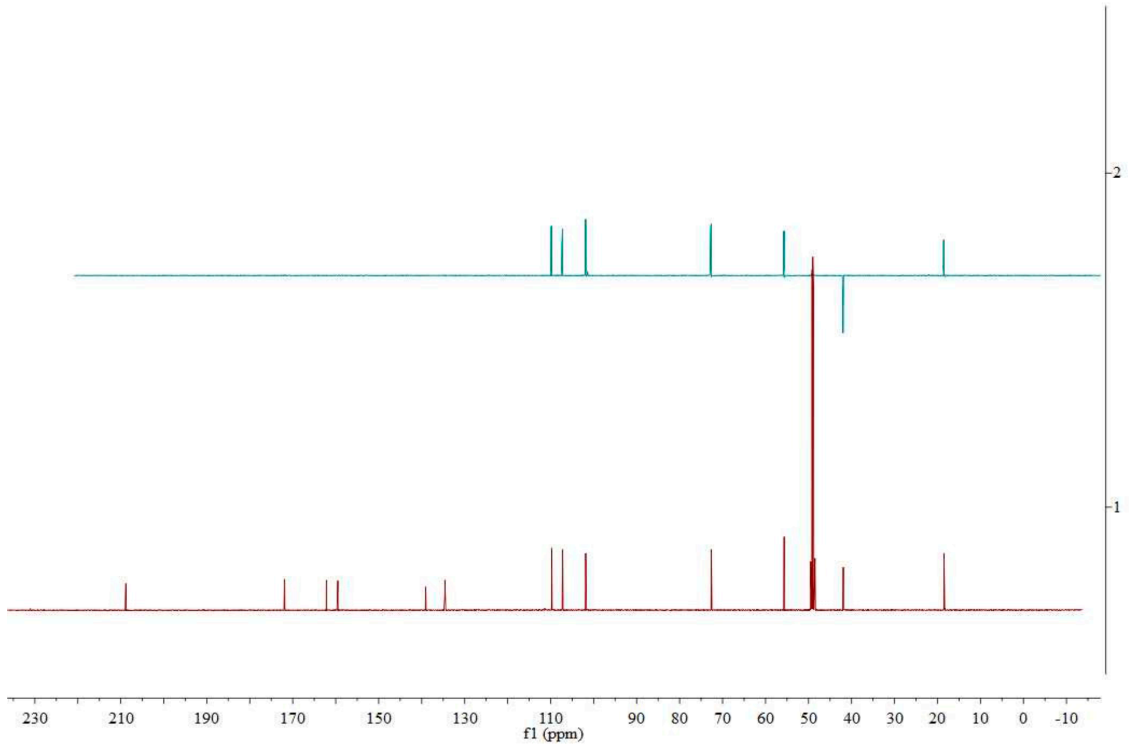

Figure S33. DEPT NMR spectrum of **6**/**7** in CD_3OD.

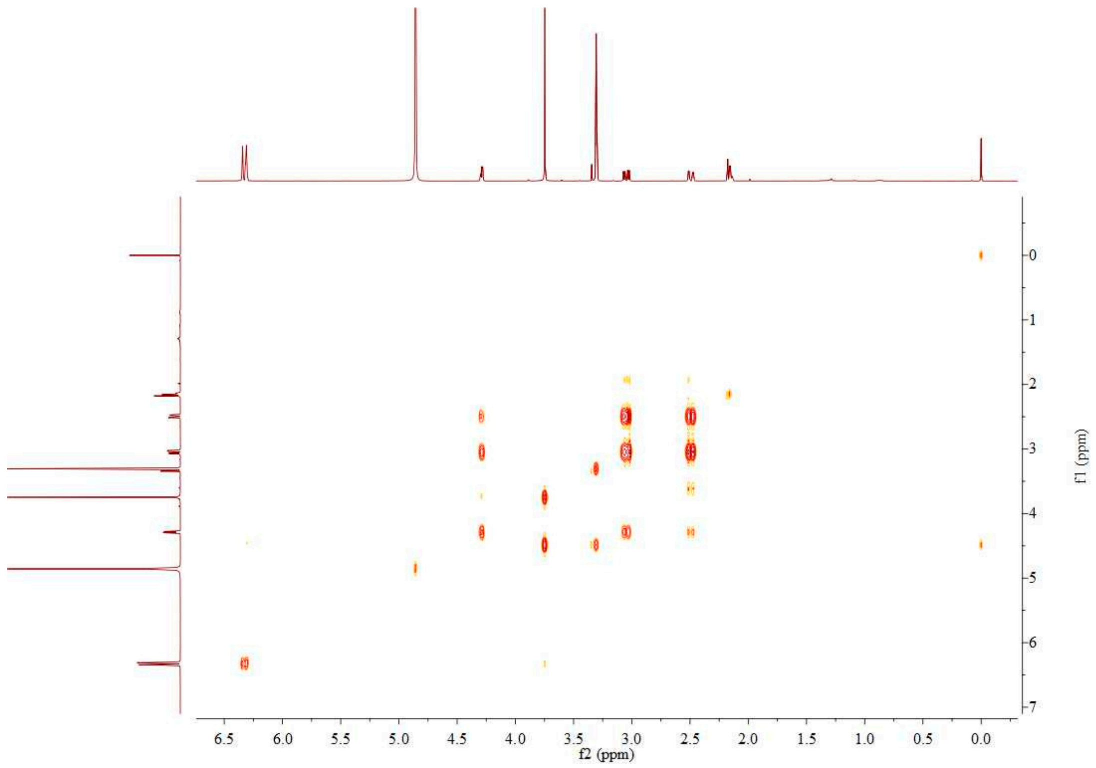

Figure S34. COSY spectrum of **6**/**7** in CD_3OD.

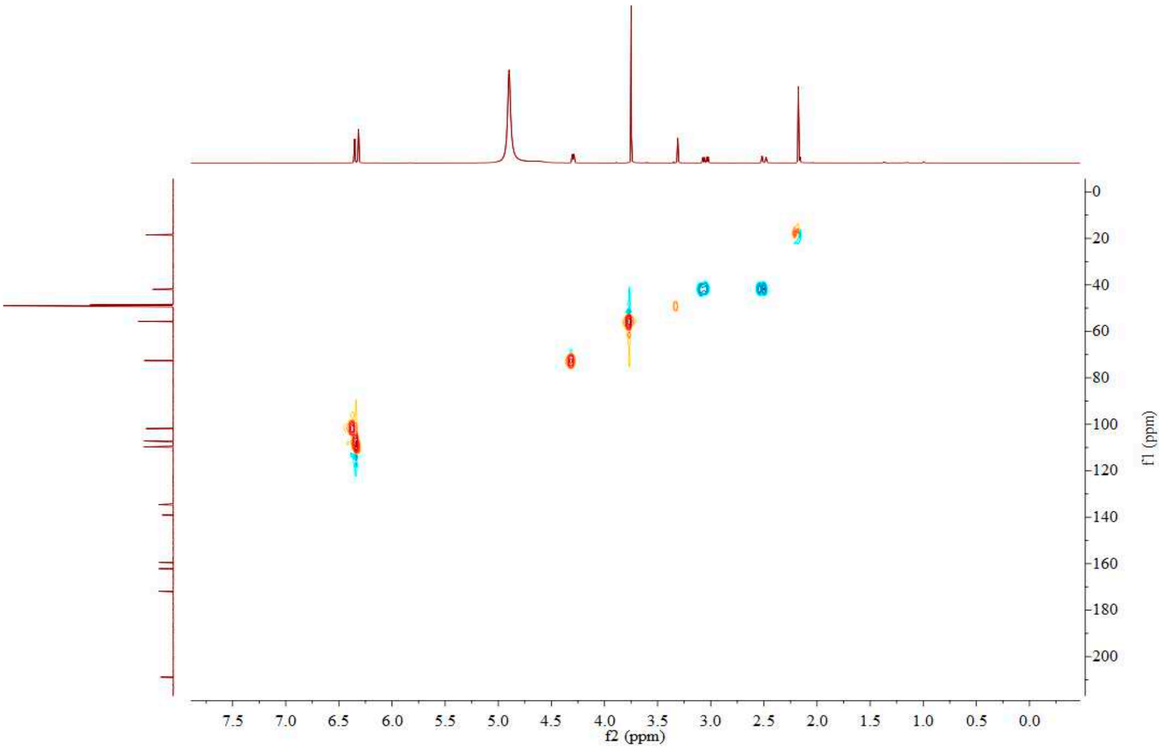

Figure S35. HSQC spectrum of **6**/**7** in CD_3OD.

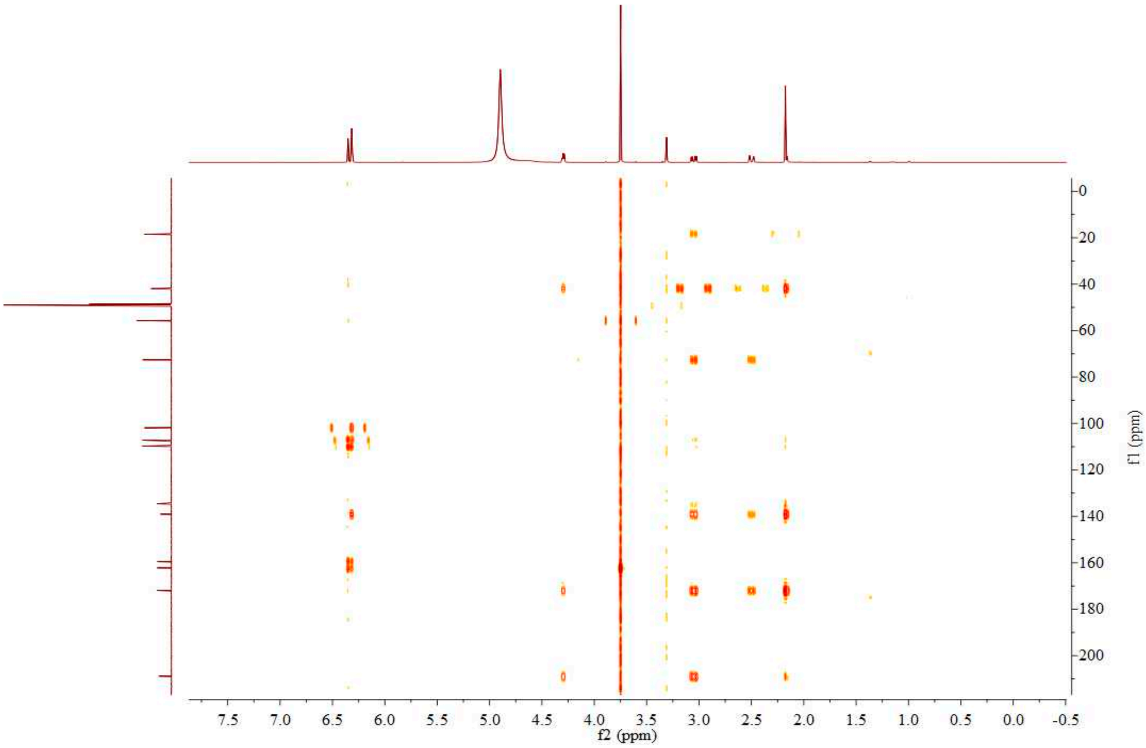

Figure S36. HMBC spectrum of **6**/**7** in CD_3OD.

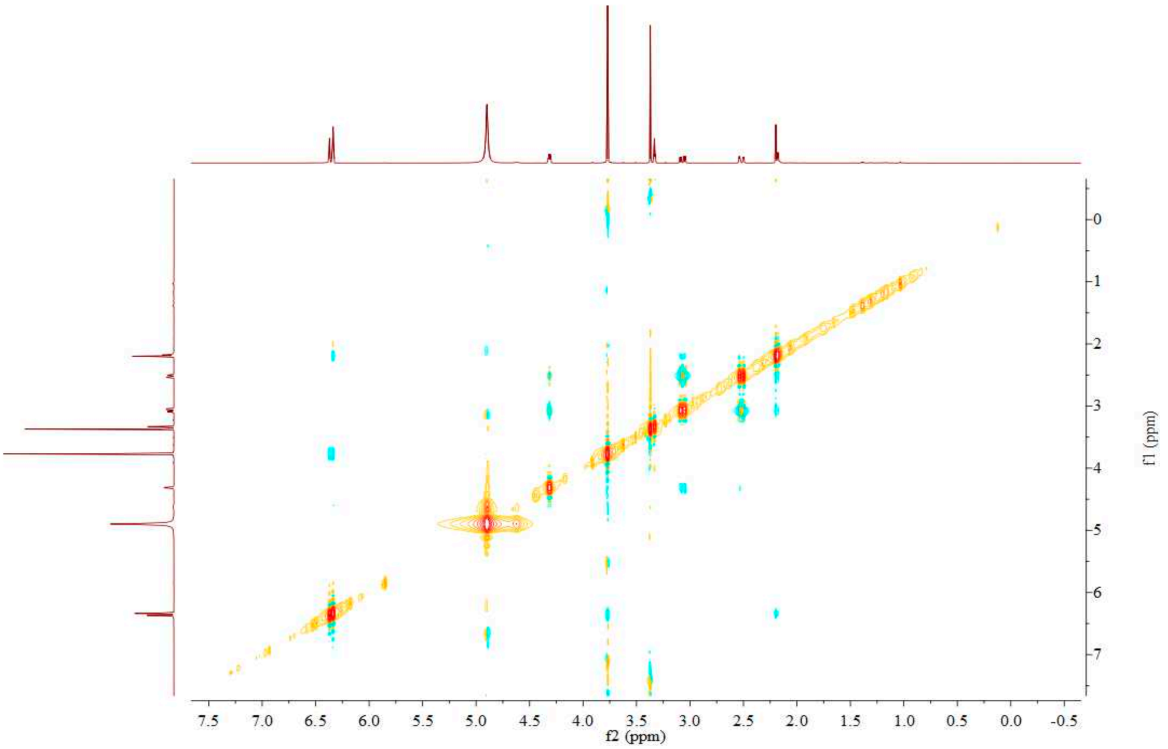

Figure S37. NOESY spectrum of **6/7** in CD_3OD.

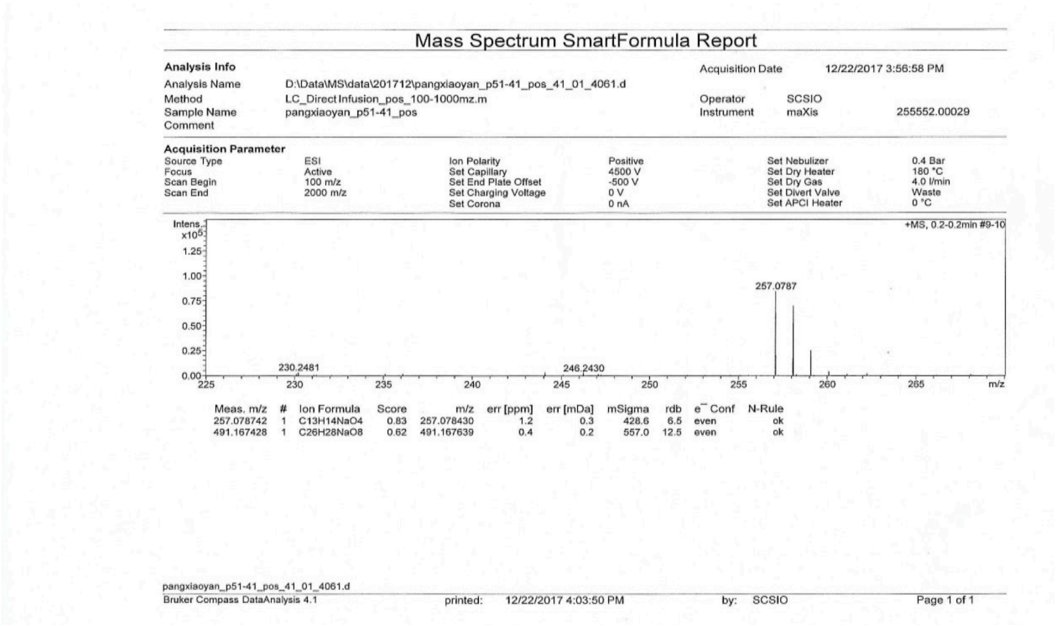

Mass Spectrum SmartFormula Report

Analysis Info

Analysis Name D:\Data\MS\data\201712\pangxiaoyan_p51-41_pos_41_01_4061.d
Method LC_Direct Infusion_pos_100-1000mz.m
Sample Name pangxiaoyan_p51-41_pos
Comment

Acquisition Date 12/22/2017 3:56:58 PM
Operator SCSIO
Instrument maXis 255552.00029

Acquisition Parameter

Source Type	ESI	Ion Polarity	Positive	Set Nebulizer	0.4 Bar
Focus	Active	Set Capillary	4500 V	Set Dry Heater	180 °C
Scan Begin	100 m/z	Set End Plate Offset	-500 V	Set Dry Gas	4.0 l/min
Scan End	2000 m/z	Set Charging Voltage	0 V	Set Divert Valve	Waste
		Set Corona	0 nA	Set APCI Heater	0 °C

Meas. m/z	#	Ion Formula	Score	m/z	err [ppm]	err [mDa]	mSigma	rdb	e⁻ Conf	N-Rule
257.078742	1	C13H14NaO4	0.83	257.078430	1.2	0.3	428.6	6.5	even	ok
491.167428	1	C26H28NaO8	0.62	491.167639	0.4	0.2	557.0	12.5	even	ok

pangxiaoyan_p51-41_pos_41_01_4061.d
Bruker Compass DataAnalysis 4.1 printed: 12/22/2017 4:03:50 PM by: SCSIO Page 1 of 1

Figure S38. HRESIMS spectrum of **6/7** in CD_3OD.

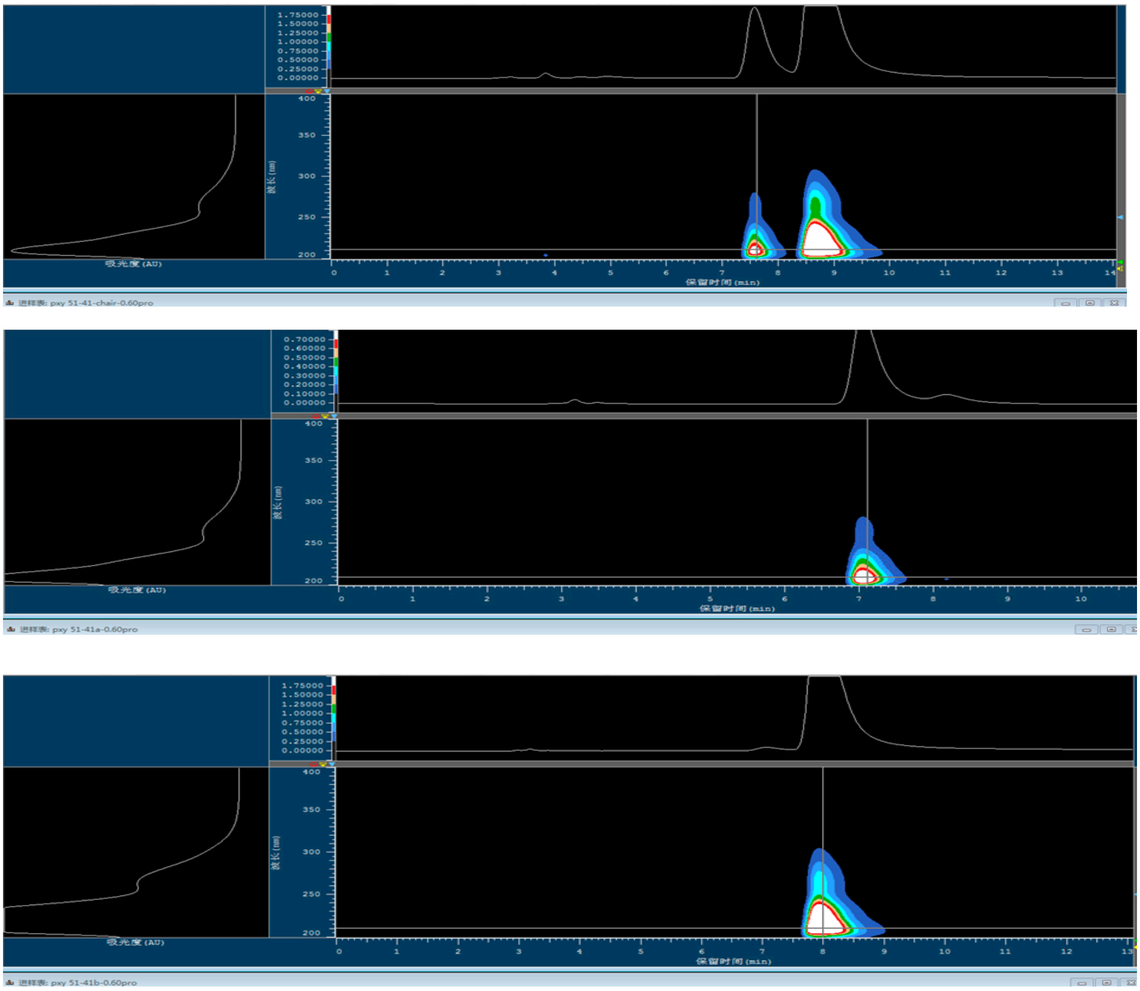

Figure S39. Chiral HPLC analyses of compounds **6** and **7** (Phenomenex Lux Cellulose-2, 4.6 mm × 25 mm, eluent n-hexane−iso-propanol, 40:60 *v*/*v*, 1 mL/min).
